# Investigation of a global mouse methylome atlas reveals subtype-specific copy number alterations in pediatric cancer models

**DOI:** 10.1038/s41588-025-02419-4

**Published:** 2025-12-11

**Authors:** Melanie Schoof, Tuyu Zheng, Martin Sill, Roland Imle, Alessia Cais, Lea Altendorf, Alicia Fürst, Nina Hofmann, Kati Ernst, Dominik Vonficht, Kenneth Chun-Ho Chan, Tim Holland-Letz, Andreas Postlmayr, Ryo Shiraishi, Wanchen Wang, Alaide Morcavallo, Michael Spohn, Carolin Göbel, Judith Niesen, Levke-Sophie Peter, Franck Bourdeaut, Zhi-Yan Han, Yanxin Pei, Najiba Murad, Fredrik J. Swartling, Jessica Taylor, Monika Yadav, Garrett R. Gibson, Richard J. Gilbertson, Matthias Dottermusch, Rajanya Roy, Kornelius Kerl, Rainer Glass, Jiying Cheng, Martin A. Horstmann, Gerrit Wolters-Eisfeld, Haotian Zhao, Dominik Sturm, Viveka Nand Yadav, Louis Chesler, Simon Haas, William A. Weiss, Paul A. Northcott, Lena M. Kutscher, Ana Guerreiro Stucklin, Olivier Ayrault, Julia E. Neumann, Daisuke Kawauchi, David T. W. Jones, Kristian Pajtler, Ana Banito, Stefan M. Pfister, Ulrich Schüller, Marc Zuckermann

**Affiliations:** 1 Research Institute Children’s Cancer Center Hamburg, Hamburg, Germany; 2 Department of Pediatric Hematology and Oncology, University Medical Center Hamburg-Eppendorf, Hamburg, Germany; 3 Mildred Scheel Cancer Career Centre HaTriCS4, University Medical Centre Hamburg-Eppendorf, Hamburg, Germany; 4 Division of Pediatric Neurooncology, Hopp Children’s Cancer Center Heidelberg (KiTZ), Heidelberg, Germany; 5 National Center for Tumor Diseases (NCT) Heidelberg, a partnership between DKFZ and Heidelberg University Hospital, Heidelberg, Germany; 6 German Cancer Research Center (DKFZ), Heidelberg, Germany; 7 Center of Excellence in Neuro-Oncology Sciences, St. Jude Children’s Research Hospital, Memphis, TN USA; 8 Department of Developmental Neurobiology, St. Jude Children’s Research Hospital, Memphis, TN USA; 9 Department of Pediatric Oncology, Hematology and Immunology, Heidelberg University Hospital, Heidelberg, Germany; 10 Institute of Neuropathology, University Medical Center Hamburg-Eppendorf, Hamburg, Germany; 11 Division of Pediatric Glioma Research, Hopp Children’s Cancer Center Heidelberg (KiTZ), Heidelberg, Germany; 12 Heidelberg Institute for Stem Cell Technology and Experimental Medicine (HI-STEM gGmbH), Heidelberg, Germany; 13 Division of Stem Cells and Cancer, German Cancer Research Center (DKFZ) and DKFZ-ZMBH Alliance, Heidelberg, Germany; 14 Department of Biostatistics, German Cancer Research Center (DKFZ), Heidelberg, Germany; 15 Translational Brain Tumor Research Group, Children’s Research Center, University Children’s Hospital Zurich, Zurich, Switzerland; 16 Department of Biochemistry and Cellular Biology, National Institute of Neuroscience, NCNP, Tokyo, Japan; 17 Division of Clinical Studies, The Institute of Cancer Research, London, UK; 18 SIREDO Pediatric Oncology Center, Institut Curie, Paris-Science Lettres University, Paris, France; 19 Center for Cancer and Immunology, Brain Tumor Institute, Children’s National Health System, Washington, DC USA; 20 Department of Immunology, Genetics and Pathology, Uppsala University, Uppsala, Sweden; 21 Cancer Research UK Cambridge Institute, Li Ka Shing Centre, University of Cambridge, Cambridge, UK; 22 Department of Pediatrics, Children’s Mercy Research Institute (CMRI), Kansas City, MO USA; 23 Center for Molecular Neurobiology (ZMNH), University Medical Center Hamburg-Eppendorf, Hamburg, Germany; 24 Department of Pediatric Hematology and Oncology, University Children’s Hospital Münster, Münster, Germany; 25 Neurosurgical Research, Department of Neurosurgery, LMU University Hospital, LMU Munich, München, Germany; 26 Department of General, Visceral and Thoracic Surgery, University Medical Center Hamburg-Eppendorf, Hamburg, Germany; 27 Department of Biomedical Sciences, New York Institute of Technology College of Osteopathic Medicine, Old Westbury, NY USA; 28 Department of Pediatrics, University of Missouri Kansas City School of Medicine, Kansas City, MO USA; 29 Department of Cancer Biology, University of Kansas Cancer Center, Kansas City, KS USA; 30 Paediatric Oncology Experimental Medicine Centre, The Institute of Cancer Research, London, UK; 31 Children and Young People’s Unit, The Royal Marsden Hospital, London, UK; 32 Berlin Institute of Health (BIH) at Charité Universitätsmedizin Berlin, Berlin, Germany; 33 Berlin Institute for Medical Systems Biology, Max Delbrück Center for Molecular Medicine in the Helmholtz Association, Berlin, Germany; 34 UCSF Helen Diller Family Comprehensive Cancer Center, San Francisco, CA USA; 35 Department of Pediatrics, University of California San Francisco, San Francisco, CA USA; 36 Developmental Origins of Pediatric Cancer Junior Research Group, German Cancer Research Center (DKFZ), Heidelberg, Germany; 37 Division of Oncology, University Children’s Hospital Zurich, Zurich, Switzerland; 38 Institut Curie, PSL Research University, CNRS UMR, INSERM, Orsay, France; 39 Université Paris Sud, Université Paris-Saclay, CNRS UMR 3347, INSERM U1021, Orsay, France; 40 Soft-Tissue Sarcoma Junior Research Group, DKFZ, Heidelberg, Germany

**Keywords:** Epigenetics, Cancer, Experimental models of disease, Genetics research, Cancer

## Abstract

Copy number alterations (CNAs) are hallmarks of cancer, yet investigation of their oncogenic role has been hindered by technical limitations and missing model systems. Here we generated a genome-wide DNA methylation and CNA atlas of 106 genetic mouse models across 31 pediatric tumor types, including 18 new models for pediatric glioma. We demonstrated their epigenetic resemblance to human disease counterparts and identified entity-specific patterns of immune infiltration. We discovered that mouse tumors harbor highly recurrent CNA signatures that occur distinctly based on the tumor subgroup and driving oncogene and showed that these CNAs share syntenic regions with the matching human tumor types, thereby revealing a conserved but previously underappreciated role in subgroup-specific tumorigenesis that can be analyzed using the presented models. Our study provides insights into globally available mouse models for pediatric solid cancers and enables access to functional CNA interrogation, with the potential to unlock new translational targets in pediatric cancers.

## Abstract (web summary)

DNA methylation and copy number variant analyses across a large number of genetic mouse models of pediatric brain tumors reveal subtype-specific molecular alterations shared with the corresponding human diseases.

## Main

Childhood tumors such as pediatric high-grade glioma (pHGG) and medulloblastoma (MB) present a formidable clinical challenge owing to their rarity, aggressiveness and intertumoral heterogeneity^1–3^. A crucial aspect of these tumors that is still elusive is the role of CNAs in cancer development^4–6^. CNAs, involving gains or deletions of large genomic segments, have been described across various cancer entities^7–10^. However, investigation of their functional role has been hindered by analyses of only fractions of heterogenous tumors, as well as a lack of patient material from the early stages of tumorigenesis^4,5^. Unraveling the function of CNAs during tumor initiation would greatly advance our understanding of tumor evolution and provide a basis for new therapeutic approaches^4–6,11^. To elucidate the underlying tumor biology and evaluate new treatment approaches, well-characterized experimental models are required^12–14^. Mouse models have emerged as the gold standard owing to their genetic similarity to humans and ability to recapitulate key aspects of tumor biology and progression^15^. However, for many disease subtypes, suitable mouse models are still missing, and even for established models, epigenetic characterization and molecular classification have rarely been performed owing to technical limitations^13,16–19^.

## Results

### Autochthonous cohort of 18 mouse models for pHGG

**Fig. 1:**
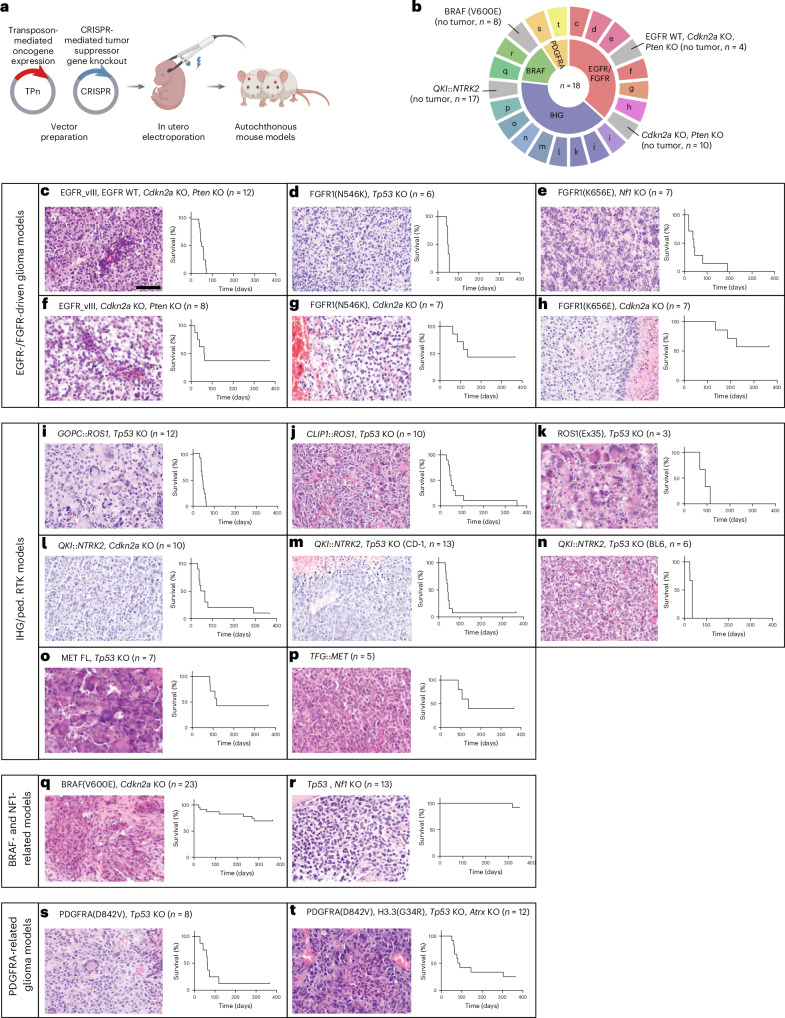
Autochthonous cohort of mouse models for pediatric glioma. **a**, Mouse models of pediatric glioma were generated by in utero electroporation of CRISPR and Tol2-transposon constructs. **b**, Eighteen different mouse models for various glioma types were generated, with tumor development in 18 of 22 electroporated constructs. Models were categorized into the indicated four subgroups (BRAF-driven, EGFR/FGFR-driven, PDGFRA-driven or IHG-like) and are color-coded respectively. Hit combinations that did not induce tumor formation are shown in gray. Letters indicate the figure panels (**c**–**t**) showing the respective model. **c**–**t**, Different hit combinations induced brain tumor formation in mice. The model latency and penetrance depended on the hits involved. Mouse tumors showed histological features of high-grade glioma including necrosis and pleomorphic nuclei, as well as mitoses indicating rapid proliferation. Scale bar, 100 μm. Panels **a** and **b** created using BioRender.com. FL, full length.

**Extended Data Fig. 1:**
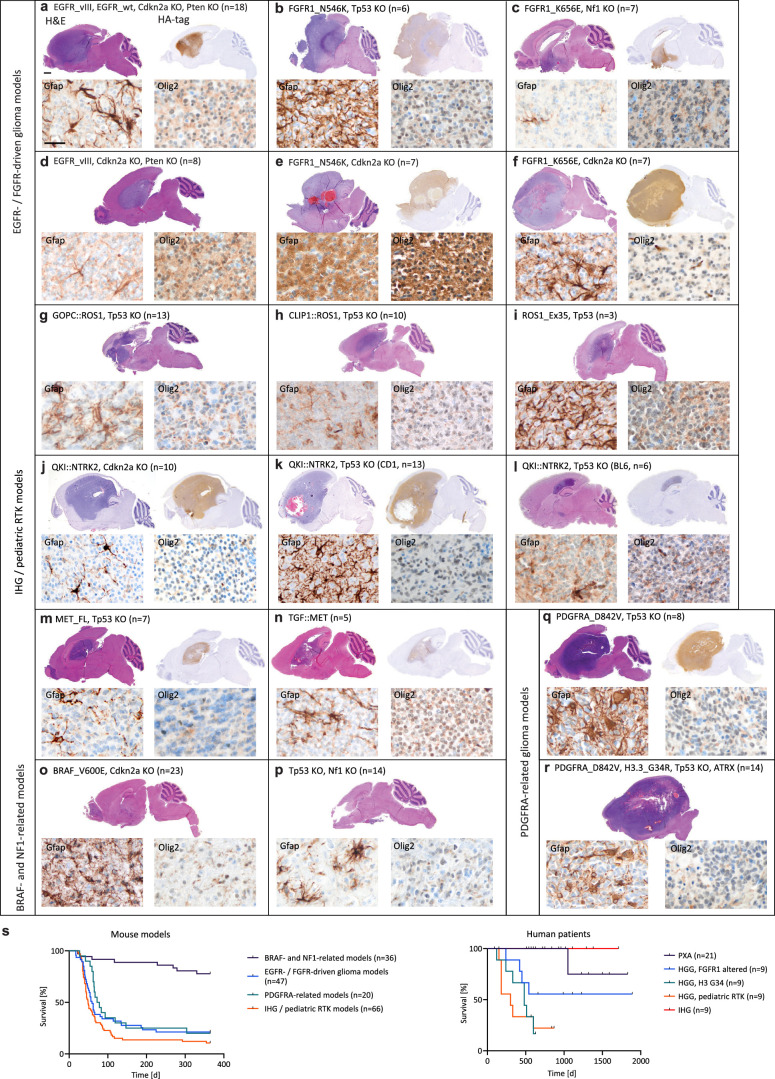
Histological overview of novel glioma mouse model cohort. **a**–**r**, Mouse models of pediatric high-grade glioma (pHGG) were generated by in utero electroporation of the indicated CRISPR and Tol2-transposon constructs. H&E overview pictures are demonstrated on the left, immunohistochemical staining of an HA-tag is displayed on the right. The staining is positive for all models with an HA-tag present in the vector. All tumors include tumor cells expressing the glial cell markers Gfap and/or Olig2. The scale bars in (**a**) represents 500 μm in all overview pictures and 100 μm in all high-magnification IHC pictures. **s**, Combined survival of mouse models and closest corresponding human entity. IHG = Infantile hemispheric glioma. FL = full length, KO = knockout, IHG = Infant High-Grade Glioma, RTK = Receptor Tyrosine Kinase.

As pHGG is among the most common malignant brain tumors in children and is characterized by vast intertumoral heterogeneity^20,21^, we initially aimed to establish a mouse model cohort representing this complexity (Fig. 1a–t). Using in utero electroporation, we delivered transposon vectors expressing pHGG-related, HA(YPYDVPDYA)-tagged oncogenes as well as CRISPR vectors inducing distinct tumor suppressor gene knockouts (KOs) at embryonic stage E14.5 to orthotopic brain regions (Fig. 1a). Of 22 tested hit combinations, 18 induced tumorigenesis and were thus used to generate new pHGG models with model-specific latencies (~20 to >100 days; Fig. 1b–t and Extended Data Fig. 1a–r). The number of mice per model varied owing to the technical variability of our somatic gene transfer method. We grouped these models into four pHGG subgroups, each representing a distinct cohort of the human disease. Despite inherent limitations of comparing the survival of untreated and genetically less complex mouse models to that of treated patients with more diverse genetic backgrounds, survival in mouse models was comparable to that in the respective human diseases, with the exception of infantile hemispheric gliomas (IHGs), for which effective therapies are available^13,22,23^ (Extended Data Fig. 1s). For most of these models, histopathological analyses revealed highly proliferative tumors showing features of human high-grade glioma including necrosis, pleomorphic nuclei, and positive staining for glial cell markers (GFAP and/or OLIG2) (Fig. 1c–t and Extended Data Fig. 1a–r).

Only 2 of 14 mice in which *Trp53* was knocked out together with *Nf1* developed tumors, after nearly 1 year (Fig. 1r). No other mice that received KO of tumor suppressor genes alone or in combination with insufficient oncogenes developed tumors; this validated the specific oncogenic potential of the remaining hit combinations (Fig. 1b and Supplementary Table 1).

We electroporated two different vectors encoding FGFR1 variants, FGFR1(K656E) and FGFR1(N546K), both of which resulted in similar tumor latencies of <100 days when paired with *Nf1* or *Trp53* KO (Fig. 1d,e). By comparison, combination with *Cdkn2A* KO increased the latency and lowered the penetrance, effects that were even more striking for the FGFR1(K656E) variant (Fig. 1g,h), consistent with the reported higher genomic instability induced by KO of *TP53* (ref. ^24^). The models related to IHG and/or pediatric receptor tyrosine kinase (RTK)-driven glioma (ped. RTK) displayed nearly full penetrances and short latencies (Fig. 1i–p), characteristics that were most reflective of the human ped. RTK group^22^ (Extended Data Fig. 1s). We previously reported a very aggressive MET-driven model after electroporation of *TFG::MET* in combination with *Trp53* KO^25^. Here we additionally tested the oncogenic potential of delivering *TFG::MET* alone or the full-length, wild-type *MET* gene in combination with *Trp53* KO. We found that the penetrance was reduced to 3/5 and 4/7, respectively, and the latency was prolonged to ~100 days (Fig. 1o,p). Delivery of a vector encoding BRAF(V600E) did not induce tumorigenesis, whereas 7 of 23 mice developed tumors when BRAF(V600E) was combined with KO of *Cdkn2a*. Notably, these tumors were much less aggressive than those of the aforementioned models, recapitulating the intermediate grade of pleomorphic xanthoastrocytoma that is typically driven by these two hits (Fig. 1q). In line with previous reports^26,27^, we generated a model driven by H3.3(G34R) coexpressing PDGFRA(D842V). However, PDGFRA(D842V) in combination with *Trp53* KO alone resulted in a similar latency and penetrance, compared to additional expression of H3.3(G34R) or KO of *Atrx*. This suggests that constitutively active PDGFRA signaling is a sufficient driver in mouse cells, irrespective of the histone mutations mentioned above or loss of *Atrx* in this setting (Fig. 1s,t), consistent with recent reports of PDGFRA as a potent driver of gliomagenesis^28^.

Thus, our comprehensive cohort of immunocompetent, autochthonous mouse models for pHGG represents an extensive resource for preclinical and tumor-biological studies.

### Methylation profiles reflect tumor entities

**Fig. 2:**
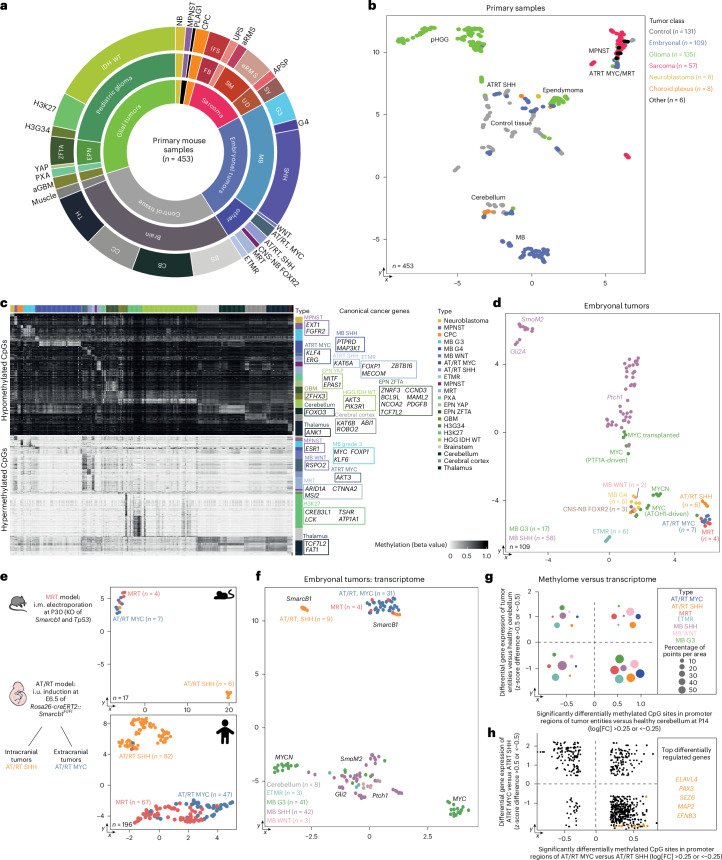
DNA-methylation-based clustering meaningfully stratifies mouse tumors. **a**, Cohort overview. **b**, UMAP of all 453 primary samples using the 10,000 most significantly differentially methylated CpG sites. **c**, Heatmap of significantly differentially methylated CpG sites in promoter regions between all entities. Canonical cancer genes are annotated. **d**, UMAP of methylation profiles of mouse models for embryonal tumors. **e**, Comparison of three different mouse models driven by loss of tumor suppressor *Smarcb1*. The model for MRTs was generated by intramuscular (i.m.) electroporation of adult mice and additional KO of *Trp53*. The models for AT/RTs were generated by embryonal tamoxifen-driven loss of *Smarcb1*. These models develop two types of AT/RT depending on tumor location. Methylation profiles of mouse rhabdoid tumor models showed more similarity between MRT and AT/RT MYC compared to AT/RT SHH tumors. The 10,000 most significantly differentially methylated CpG sites are displayed. The corresponding human entities showed the same pattern in their methylation profile, with MRTs and AT/RT MYCs clustering in proximity. **f**, UMAP of integrated gene expression data of publicly available GEO datasets (for details, see Methods) of embryonal mouse tumor models. **g**, Comparison of transcriptome and methylome data. The average of each tumor entity was compared to data for healthy cerebellum, and the *z*-score (for transcriptome) or log fold change (for methylome) was calculated. After applying a cutoff (*z*-score difference >0.5 or <−0.5 and log fold change >0.25 or <−0.25), XY-plots were constructed, and the number of points per area was calculated. **h**, XY-plot of gene expression and DNA methylation of ATRT MYC versus ATRT SHH. Genes that were also significantly differentially expressed in human ATRTs are shown in orange (Extended Data Fig. 2i). NB, neuroblastoma; MPNST, malignant peripheral nerve sheath tumor; CPC, choroid plexus carcinoma; FB, fibroblastic tumors; IFS, infantile fibrosarcoma; UPS, undifferentiated pleomorphic sarcoma; SM, skeletal muscle tumor; aRMS, alveolar rhabdomyosarcoma; eRMS, embryonal rhabdomyosarcoma; UD, uncertain differentiation tumors; APSP, alveolar soft part sarcoma; Sy, synovial sarcoma; ETMR, embryonal tumor with multilayered rosettes; aGBM, adult glioblastoma; PXA, pleomorphic xanthoastrocytoma; EPN, ependymoma; WT, wild type; BS, brainstem; CB, cerebellum; CC, cerebral cortex; TH, thalamus; FC, fold change; CNS-NB, central nervous system neuroblastoma; i.u., in utero; G3, grade 3.

**Extended Data Fig. 2:**
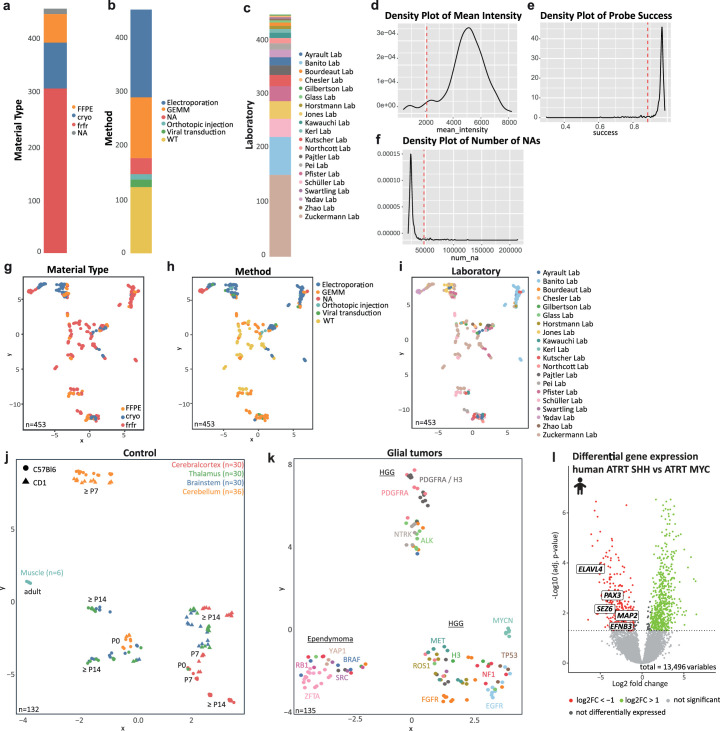
Composition of the sample cohort. **a**–**c**, Samples from three different material types, >5 different methods for mouse model generation and 20 different laboratories were included. **d**–**f**, Quality control assessment leads to the exclusion of 19 samples. Exclusion criteria are mean intensity, number of missing values (NAs) and probe success. For all criteria, the 95th percentile was calculated, and all samples in the lowest 5% in all three filter criteria were excluded for all further analyses. **g**–**i**, UMAPs of the primary samples show no material-, method- or lab-specific methylation pattern. **j**, UMAP-analysis of control samples reveals a tissue, age and strain dependent methylation profile. **k**, UMAP-analysis of glial tumor samples shows oncogene-dependent methylation profiles. **l**, Volcano plot of differential gene expression between human ATRT SHH and human ATRT MYC. In red are genes which have a log2-fold change >1 and are expressed mainly in ATRT SHH, in green are the genes significantly higher expressed in ATRT MYC. Labeled are genes also found in mouse samples showing high correlation between RNA and methylome (Fig. 2h). Significantly expressed genes were determined by a two-sided t-test and values were corrected for multiple testing by the Benjamini-Hochberg procedure.

With the aim of extending our mouse model platform to a representative DNA methylome atlas of pediatric solid tumor mouse models, we formed a global collaboration and collected 567 samples, of which 548 fulfilled quality criteria (consisting of 453 primary tissue samples with an average of 3 samples per model or control tissue and 15 sorted immune cell populations, as well as 80 passaged tumor samples) representing 106 different mouse models from 20 laboratories^10,13,16–18,29–50^ (Fig. 2a and Extended Data Fig. 2a–c). Our comprehensive tumor cohort comprised 7 families (glioma, embryonal tumors, neuroblastoma, sarcoma, choroid plexus tumors, control tissues and others), 15 classes and 31 tumor types. Initially, we performed quality control and filtered samples based on established quality metrics of DNA methylation arrays (number of missing values, mean probe intensity and probe success; Extended Data Fig. 2d–f). UMAP visualization of primary samples indicated that grouping was based on the respective class (Fig. 2b) and to a lesser extent according to tissue material, mouse model establishment method or origin laboratory (Extended Data Fig. 2g–i), fostering comparisons between different samples.

Analysis of differentially methylated CpG sites in promoter regions between tumor types highlighted tumor-driving oncogenes^51^ such as *MYC* in MB grade 3 but also revealed potential drivers that have only recently been brought into context with the respective entity, such as *PDGFB* for EPN ZFTA^52^ or *PIK3R1* for IDH wild-type glioma^53^ (Fig. 2c).

Next, we compared the methylation profiles of control samples, and observed a grouping according to the brain region they had been isolated from (Extended Data Fig. 2j). The impact of mouse age on the methylation profile was smallest at postnatal day 0, consistent with previous studies showing that the mouse brain further matures and differentiates after birth^54^.

For glial tumors, the driving oncogene was the main determinant of sample clustering, with ependymoma being distinct from pHGG, as expected (Extended Data Fig. 2k). Notably, NTRK- and ALK-driven tumors formed a cluster separate from MET- and ROS1-driven gliomas, indicating systematic differences between these two groups with similar oncokinases.

The clustering of embryonal tumors was according to their tumor type (Fig. 2d). Both atypical teratoid rhabdoid tumor (AT/RT) models were based on a *Rosa26-creER*^*T2*^-driven KO of *Smarcb1* at embryonal day 6.5. The malignant rhabdoid tumor (MRT) model, by comparison, was generated by KO of *Smarcb1* and *Trp53* at postnatal day 30 in cells of the thigh muscle (Fig. 2e). Despite these differences in model generation, we observed close proximity of AT/RT MYC and MRT compared to AT/RT SHH, which was readily recapitulated in the respective human counterparts (Fig. 2e). This indicates that both entities might originate from the same cellular lineage and supports previous reports suggesting a potentially shared very early cell of origin in both human and mouse^49^. The same grouping was validated in our analysis of transcriptomic data (Fig. 2f). We then compared our methylome atlas to the transcriptome of all models for which these data were available and, as expected, found the strongest differential CpG site methylation in promoter regions of genes with low expression (Fig. 2g). Of note, when performing the same analysis specifically for both ATRT subgroups, we found five genes to be differentially methylated and expressed (Fig. 2h); we also identified these genes in a differential gene expression analysis of the human subgroups (Extended Data Fig. 2i).

### Mouse models recapitulate human tumors epigenetically

**Fig. 3:**
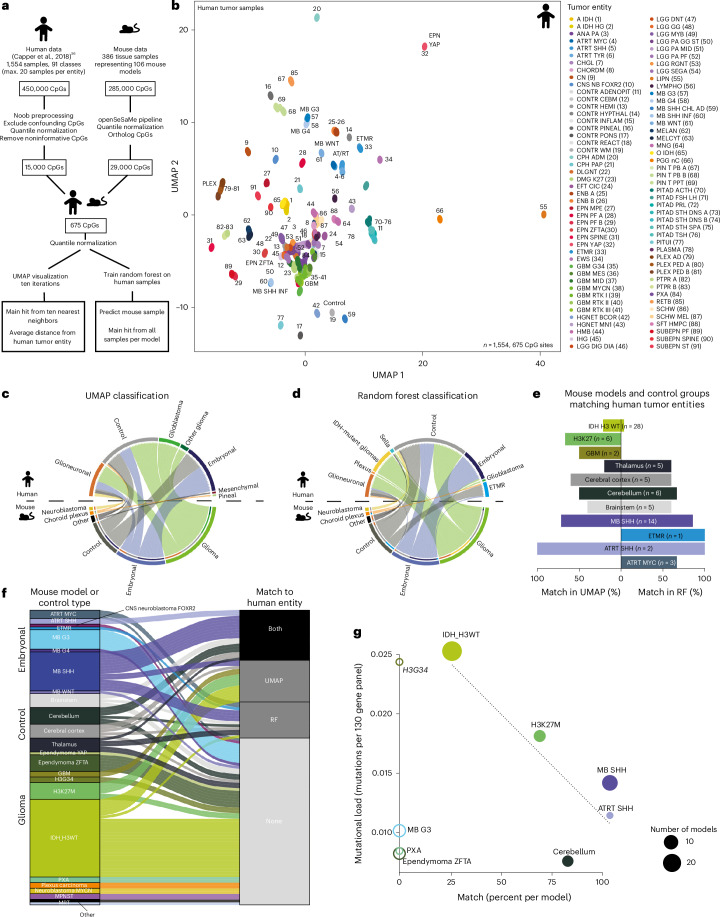
Epigenetic resemblance of mouse models to their human counterparts. **a**, Schematic of the workflow to compare mouse and human DNA methylation profiles. The human reference set was derived from Capper et al.^55^ and included 1,554 samples (a maximum of 20 samples per entity). After preprocessing, the 15,000 most significantly differentially methylated CpG sites were extracted. Of these, the 675 ortholog CpG sites on the mouse array were identified and used for analyses. **b**, UMAP of human tumor samples indicated robust clustering using only the identified 675 orthologue CpG sites. **c**, Each mouse sample was separately compared to a randomly chosen 90% of the human reference set, and this process was iterated ten times. The closest human match was calculated. **d**, A random forest (RF) classifier was trained on the same 675 CpG sites, and the entity of all mouse samples was predicted. **e**, Comparison of matching between UMAP and RF for all models that matched the expected corresponding human entity. **f**, Matching to the expected human tumor entity is displayed. Samples displayed a match in both RF and UMAP, only UMAP or only RF, or not at all. **g**, The mutational load of modeled human entities was derived from Sturm et al.^22^ and compared to the percentage of matching per model. Only entities with more than three samples in the human reference set and with more than one mouse model were included. The trend line represents a regression curve of all entities with at least one match (full circles).

**Extended Data Fig. 3:**
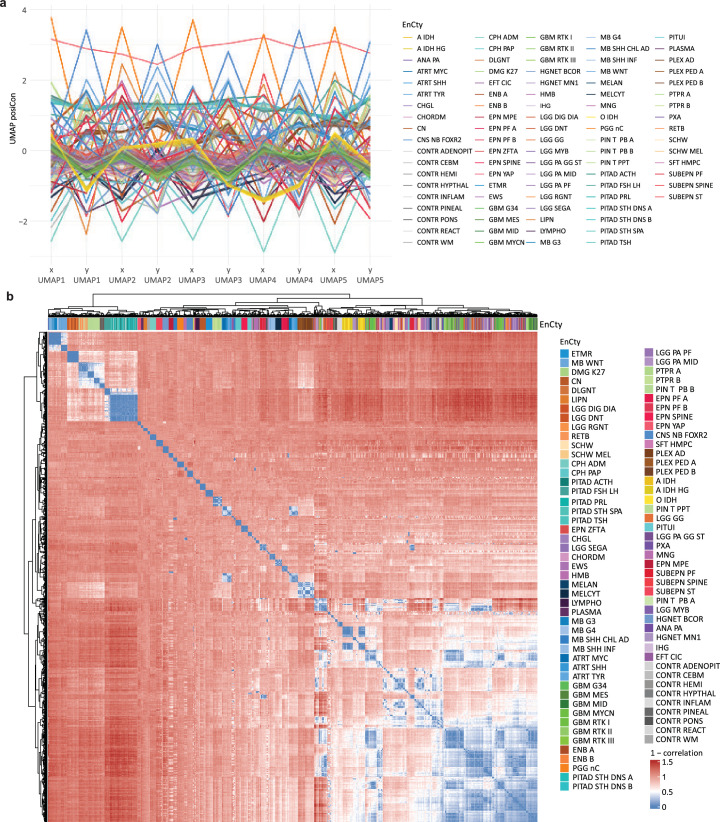
The 675 CpG sites employed for human mouse comparisons are sufficient to distinguish human CNS tumor entities. **a**, Position of each human sample in a UMAP upon mutiple iterations. Each sample is depicted as a line representing the coordinates. The resulting plot indicates that samples of one entity group together consistently validating the stability of the UMAPs. **b**, The position of each sample in 500 UMAP iterations was used to calculate the correlation of different samples. The heatmap of pearson correlation coefficients shows stable classes of human tumors representing the different entities. The correlation within classes is 0.897. This analysis shows that the selected 675 CpGs are sufficient for distinguishing human CNS tumors.

**Extended Data Fig. 4:**
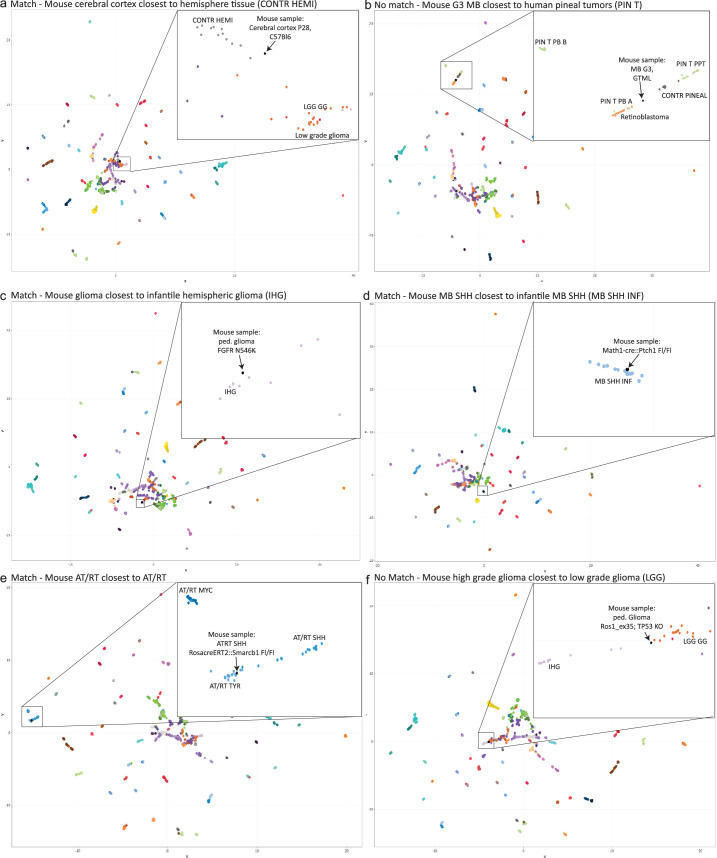
Comparing mouse methylation patterns with human tumors reveals high resemblance of mouse models to their human counterparts. **a**–**f**, Example UMAPs of DNA methylation patterns of the human reference set and one mouse sample per UMAP. Samples are color-coded based on their entity, the black arrow points at the mouse sample. The inset is a zoom into the region of the mouse sample. The abbreviations are adopted from Capper et al., 2018.

**Extended Data Fig. 5:**
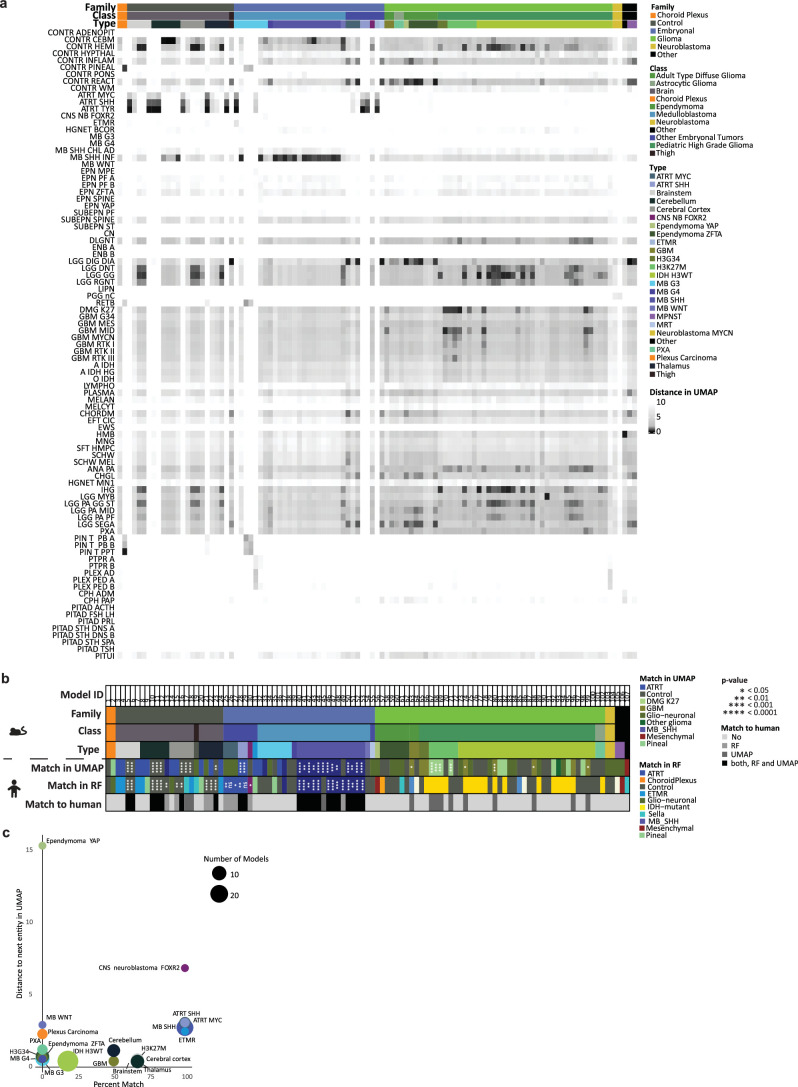
Mouse models match corresponding human entities with statistical significance. **a**, Heatmap of the average distance of each mouse model to the different human entities. **b**, Oncoplot showing the predicted entity as well as the expected human entity to each mouse model. The matches in UMAP and random forest are statistically validated using a binomial test. **c**, Dotplot showing no correlation between matching success and distinctiveness of human entities in UMAP depictions. The color legend of Familiy, Class and Type in **a**) also applies to **b**) and **c**).

We next aimed to compare the epigenome of mouse brain tumors to their human counterparts and develop a customized workflow allowing robust cross-species comparison (Fig. 3a). As a human reference set, we used the 15,000 most significantly differentially methylated CpG sites from a subset of the Heidelberg Classifier^55^. Of these 15,000 sites, 675 ortholog CpG sites were present on the mouse array (Supplementary Table 2). To validate that this small number of CpG sites was sufficient to correctly stratify human brain tumors, we visualized all human tumor samples using these 675 sites and found that different tumor classes were still readily distinguishable (Fig. 3b). We further showed that this result was stable throughout numerous UMAP iterations (Extended Data Fig. 3). Next, we performed the same UMAP depiction while adding one mouse model to the analysis cohort at a time. As expected, most normal mouse brain samples showed the highest similarity to human control tissue (Fig. 3c and Extended Data Fig. 4a), and mouse tumor families resembled human tumor classes reliably (Fig. 3c, Extended Data Fig. 4b–f and Supplementary Table 3). For further validation, we performed an independent random forest classification using a newly trained classifier based on the overlapping 675 probes and observed similarly good correlation for tumor families (Fig. 3d). Analyzing tumor types more specifically, we identified various connections. For example, the closest matches of mouse G3 MB samples were either human SHH MB, control tissue or pineal tumors (Extended Data Fig. 5a); this was in line with previous reports indicating a close connection between G3 MB and pineal tumors^56,57^ and potentially consistent with diverging cells of origin for human G3 MB and mouse tumors supposedly modeling this entity^58^. On this more granular level, only SHH MB, AT/RT SHH and control tissues most closely matched their human counterparts using both methods (Fig. 3e,f), and these predicted matches were found to be highly significant (Extended Data Fig. 5b and Supplementary Tables 4–6). As AT/RTs formed more distinct clusters in our newly generated UMAP than other entities (Fig. 3b), we confirmed that this technical distinction did not predispose a higher matching chance (Extended Data Fig. 5c). Based on these findings, we attempted to identify the discriminating principles behind mouse–human similarities. We found an inversely correlated trend of model similarities to the average mutational load of the respective human entity (Fig. 3g), suggesting that early embryonal tumors are more similar to genetically less complex mouse models than to entities that have undergone a longer period of tumor evolution. We excluded tumor types with 0% match from this analysis as most of these either involved fusion oncogenes, which likely cause different effects in mouse and human cells (H3G34/EPN ZFTA) or potentially had different cells of origins in the two species (MB grade 3 (ref. ^58^)).

### Tumors display distinct immune cell infiltration

**Fig. 4:**
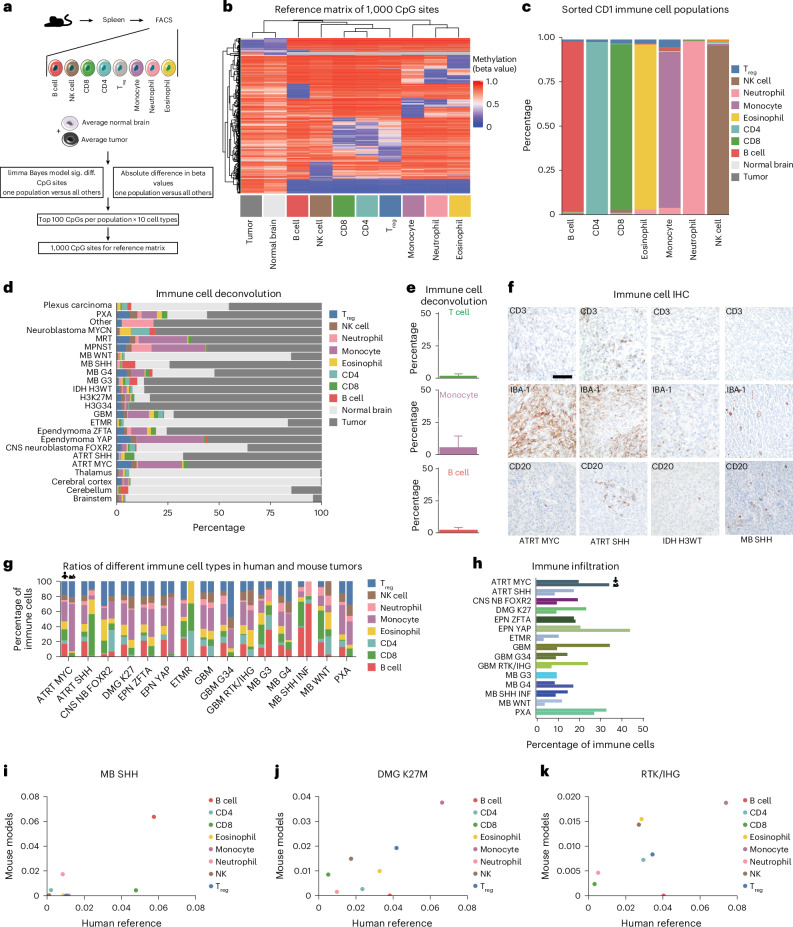
Deconvolution reveals model-specific immune infiltration. **a**, Mouse immune cells were isolated from the spleens of C57BL/6 mice, followed by FACS. Methylation profiles of B cells, NK cells, CD8 T cells, CD4 T cells, T_reg_ cells, monocytes, neutrophils and eosinophils, as well as average methylation profiles of healthy mouse brain and representative mouse brain tumors, were used to build a reference matrix of 1,000 CpG sites. **b**, Heatmap showing the reference matrix. **c**, Validation of reference-matrix-based deconvolution using FACS-sorted immune cells of CD1 mice. **d**, Deconvolution of mouse brain tumors and control brain samples revealed distinct populations of immune cells. **e**, Average percentages of T cells and B cells as well as monocytes after deconvolution. *n* = 24 in all graphs, representing 24 different tumors. Data are presented as mean values, with error bars representing the standard deviation of a two-sided *t*-test. **f**, IHC validation of the deconvolution results showing low B cell and T cell levels, as well as various monocyte infiltrations. All stainings were performed under standard conditions in at least three biological replicates. One representative picture is provided per tumor type. Scale bar, 100 μm. **g**, Comparison of immune cell compositions of mouse and human tumor samples. Deconvolution of human tumors was performed by applying the reference matrix described by Grabovska et al.^64^ to the human reference set derived from Capper et al.^55^. **h**, Comparison of immune cell ratios in human and mouse tumor samples. **i**–**k**, Correlation of mouse and human immune cell fractions in MB SHH (**i**), DMG K27M (**j**) and RTK/IHG (**k**). Sig. diff., significantly different; chr., chromosome; NS, not significant.

**Extended Data Fig. 6:**
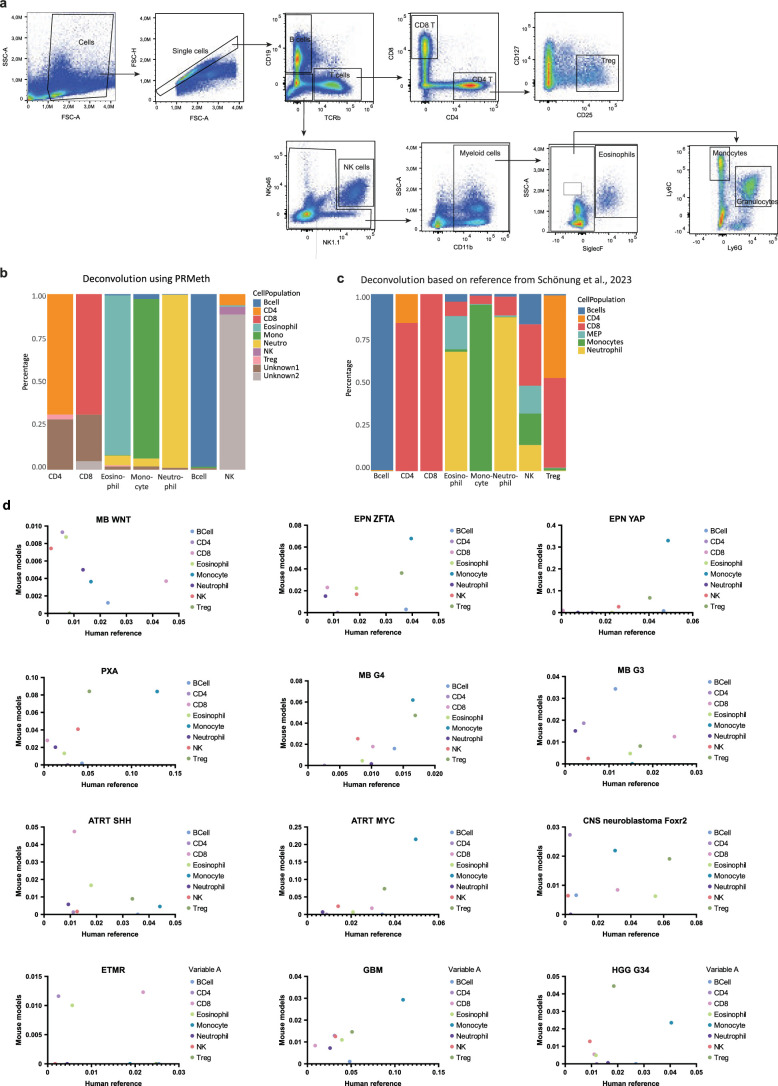
Technical validation of the deconvolution approach and comparison to human tumor samples. **a**, Gating strategies used for sorting B cells (CD45 + CD19+ TCRb-), CD4 + T cells (CD45 + CD19+ TCRb+ CD4 + CD8-), CD4+ Treg cells (CD45 + CD19- TCRb+ CD4 + CD8- CD127- CD25+), CD8 + T cells (CD45 + CD19- TCRb+ CD4- CD8+), NK cells (CD45 + CD19- TCRb- CD11bdim NKp46 + NK1.1+), Granulocytes (CD45 + CD19- TCRb- CD11b + Ly6G + Ly6C-), Monocytes (CD45 + CD19- TCRb- CD11b + Ly6G- Ly6C+) and Eosinophils (CD45 + CD19- TCRb- CD11b+ SiglecF+ SSChigh) from C57BL6 and CD1 mice. **b**, Deconvolution of the sorted mouse immune cell populations used for the newly developed reference matrix using the PRmeth algorithm39 does not show a better performance than the employed EpiDish-algorithm. **c**, To test the newly generated reference matrix for deconvolution, a published reference matrix for mouse immune cells40 was used for deconvolution of the samples of the here described reference matrix. The published reference does not include brain tissue and is not able to outperform the here described new reference matrix. **d**, Comparison of immune cell populations derived from DNA methylation deconvolution of murine and human tumors.

To investigate the landscape of immune cell infiltration across mouse models, we initially isolated various immune cell populations from C57BL/6 mice using fluorescence-activated cell sorting (FACS; Extended Data Fig. 6a) and analyzed their methylation signatures. We then generated a reference matrix by identifying the top 100 differentially methylated CpG sites of each cell population versus all others (Fig. 4a). We focused on these eight different immune cell populations, the average tumor signature of a representative subset of tumor samples and the average signature of all normal brain tissues. Thus, the reference set comprised 1,000 CpG sites displaying distinct methylation patterns across the analyzed cell populations (Fig. 4b and Supplementary Table 7). We validated this reference matrix by isolating the same immune cell populations from CD1 mice and performing deconvolution of their methylome profiles. Each immune cell population was distinctly classified and predicted to be of high purity (>94%, with monocytes as the least pure cell population; Fig. 4c), indicating that our deconvolution approach based on EpiDISH^59^ is feasible and provides faithful prediction of immune cell compositions. Given the limitations that may arise when using a mixed tumor cell reference ^60,61^, we compared this approach to deconvolution with unknown cell populations via PrMeth^62^, which provided less reliable results (Extended Data Fig. 6b). In addition, comparison to a previously published reference set^63^ indicated that our newly generated reference matrix provided a more robust basis for our dataset (Extended Data Fig. 6c). Therefore, we applied the EpiDISH deconvolution workflow using our new reference matrix to our mouse model cohort (Fig. 4d and Supplementary Table 8). As expected, the majority of normal brain tissues were predicted to comprise mostly normal brain cells, whereas a subset of cerebellar tissues was predicted to harbor up to 21% of tumor cells, highlighting the aforementioned well-described limitations of using averages of highly heterogenous tumor samples as input^60,61^.

**Extended Data Fig. 7:**
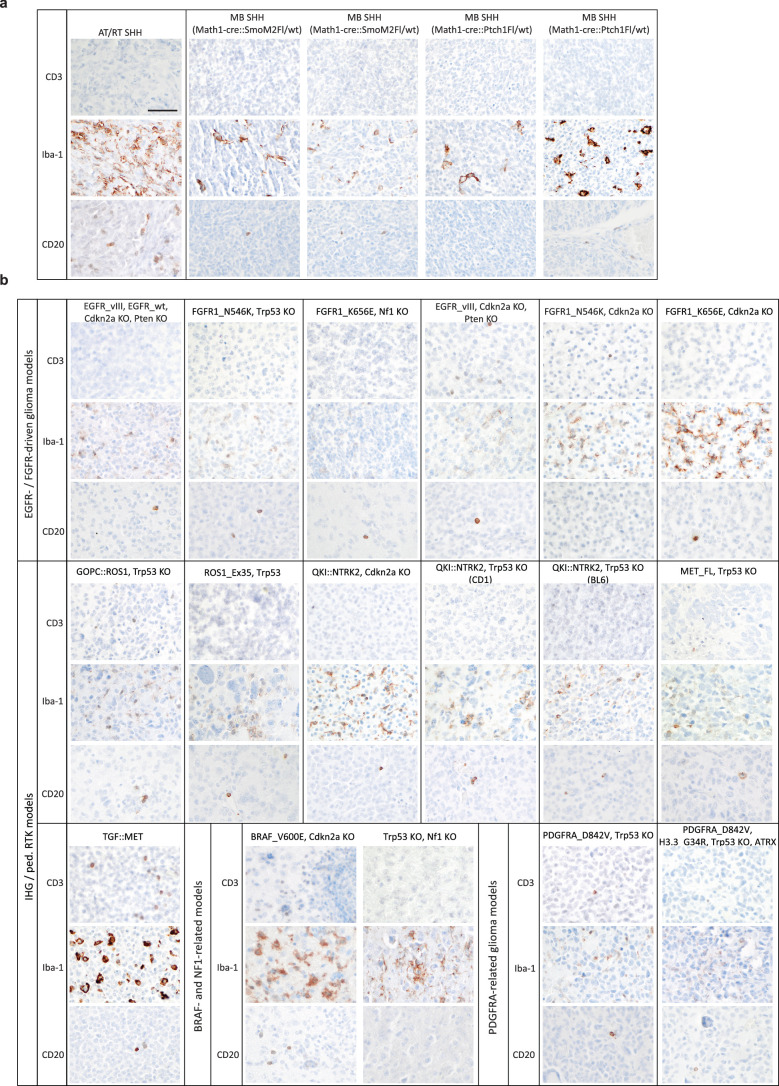
Immunhistochemistry of immune cell markers in mouse CNS tumors. **a**, T cells (CD3), monocytes (Iba-1) as well as B cells (CD20) were immunohistochemically stained in represenative embryonal tumors (AT/RT SHH and MB SHH) with available FFPE-tissue. **b**, The same immune cell markers were used to identify immune cell populations in all new glioma models presented in this study. Most samples show low to no T cell and B cell infiltration and the amount of Iba-1 positive monocytes varies among models. Scale bar represents 100 μm. All stainings in a and b were performed under standard conditions and all performed stainings are depicted.

Nevertheless, given the much more clearly defined immune cell signatures in our reference matrix, we identified various immune cell profiles in different brain tumor entities. Using immunohistochemistry (IHC), we validated the general trend of low B cell and T cell infiltration in our mouse models, as well as heterogenous macrophage levels (Fig. 4e,f and Extended Data Fig. 7). We also found various levels of normal brain cells, potentially resulting from normal tissue contamination. Compared to other brain tumor types, SHH MB was not predicted to comprise monocytes, regulatory T (T_reg_) cells or natural killer (NK) cells but instead substantial numbers of B cells (up to 8%), consistent with studies investigating human counterparts^64^. Next, we leveraged a human dataset^64^, performed a cross-species comparison and identified generally comparable immune profiles between mouse and human tumors, with a few entities such as GBM displaying nearly identical immune compositions (Fig. 4g,h). One-to-one comparisons revealed that the same specific cell type was often overrepresented in distinct entities of both species, such as B cells in SHH MB and monocytes in aggressive glioma subtypes (Fig. 4i–k and Extended Data Fig. 6d), further validating the fidelity of these models.

### Mouse models reveal subtype-specific human syntenic CNAs

**Fig. 5:**
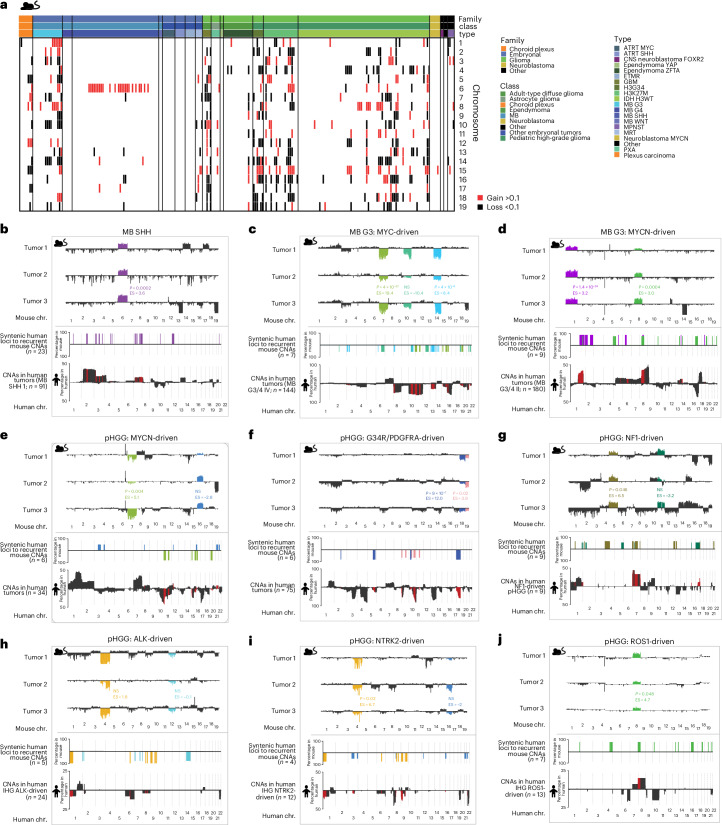
Highly recurrent mouse CNA profiles recapitulate human counterparts. **a**, Overview of identified CNAs in all mouse models. **b**–**j**, CNA profiles of three individual mouse SHH MB (**b**), MYC-driven Gr3 MB (**c**), MYCN-driven Gr3 MB (**d**), MYCN-driven pHGG (**e**), G34R/PDGFRA-driven pHGG (**f**), NF1-driven pHGG (**g**), ALK-driven pHGG (**h**), NTRK2-driven pHGG (**i**) or ROS1-driven pHGG (**j**). Recurrent changes are color-coded. Middle sections of panels display the frequency of the color-coded CNAs in all analyzed mice of that model, as well as the respective syntenic regions on the human genome. Lower sections show human CNA summary profiles of the indicated human tumor entity. Sections that did not overlap with the indicated mouse CNAs are shown in black. Syntenic overlap of each color-coded mouse CNA with human summary plots is depicted in red and was statistically analyzed as indicated by the *P* values next to the respective CNA. The *P* values were derived by one-sided *t*-tests without adjustment for multiple testing. Enrichment scores were calculated by comparing the overlap of the indicated CNA with the related human tumor entity to the overlap of that CNA with a pediatric reference set.

**Extended Data Fig. 8:**
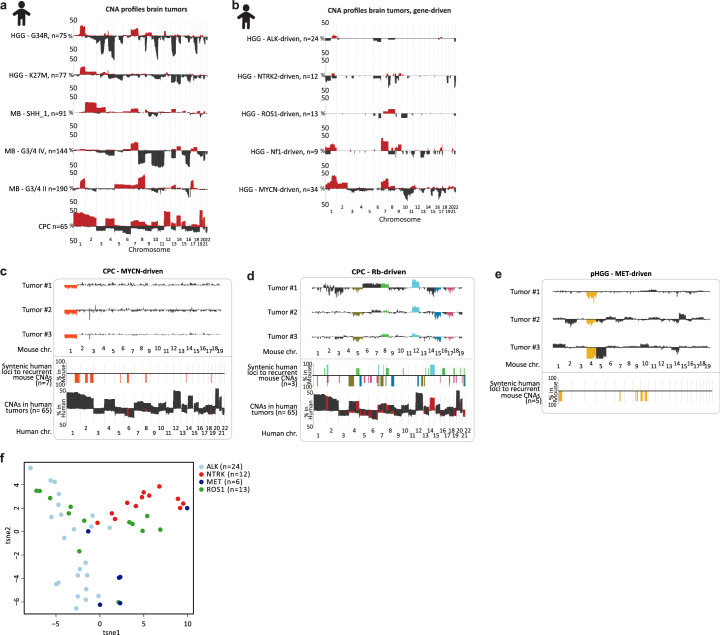
Additional CNA profiles. **a**, **b**, Cumulative CNA plots of human entities which are used for comparison to mouse tumors. **c**–**e**, CNA profiles of three individual mice per mouse model indicated. Recurrent changes are color coded. Middle sections of panels display the frequency of the color-coded CNAs in all analyzed mice of that model as well as the respective syntenic regions on the human genome. Lower sections show the average human CNA profile of the indicated human individual mice of the indicated models. Overlapping changes between mice and human in the same direction are depicted in red. **f**, T-SNE analysis of all human RTK-driven HGGs shown in Extended Data Fig. 8b, e. chr. – chromosome, CNA - copy number aberration, CPC - choroid plexus carcinoma.

Next, we analyzed the CNAs of individual tumors and found that acquired CNAs were specific to the respective tumor type (Fig. 5a). This suggests that delivered tumor-inducing hit combinations are not entirely sufficient for full-blown tumor development but that additionally acquired tumor-type-specific CNAs play a part in tumorigenesis, consistent with recent reports highlighting the importance of CNAs during tumor development^4–6,65,66^. Next, to investigate how mouse tumor CNAs matched those in human cancers, we generated CNA summary plots of specific human tumor groups that were based on either reference datasets^55^ (Extended Data Fig. 8a) or distinct tumor-driving oncogenic hits (Extended Data Fig. 8b).

Next, we analyzed the synteny of human and mouse CNAs. After plotting individual mouse copy number profiles, we again observed highly reproducible, tumor-type-specific and even oncogene-specific occurrence of mouse CNAs (Fig. 5b–j). Mouse SHH MB displayed a highly recurrent gain of chromosome 6 across different methods and different laboratories in which these models have been established. Among the four methylation subtypes of human SHH MB, subtype 1 harbored frequent gains of chromosomes 2, 3 and 7, all of which overlapped with regions syntenic to mouse chromosome 6 (*P* = 0.0002; Fig. 5b). Our MYC-driven MB models, on the other hand, were strongly characterized by recurrent losses of chromosomes 7 and 14, which significantly overlapped with most recurrent losses in subtype IV of human grade 3/4 MB (*P* = 4 × 10^−32^ and *P* = 4 × 10^−8^; Fig. 5c). In closely related MB models driven by MYCN instead of MYC, the CNA pattern was entirely different, and mouse MYCN-driven MB harbored recurrent gains of chromosomes 1 and 8 (Fig. 5d). This was recapitulated by subtype II of human group 3/4 MB, which was also characterized by syntenic gains of chromosomes 1 (*P* = 1.4 × 10^−24^) and 8 (*P* = 0.0004). PHGG models driven by MYCN displayed loss of chromosome 7, which was reflected by loss of chromosome 10 and 11 in the human summary plots (*P* = 0.004; Fig. 5e). By contrast, choroid plexus carcinoma models driven by MYCN showed a relatively flat genome with recurrent loss of chromosome 1, which did not match the severely unbalanced genomes of human choroid plexus carcinoma (Extended Data Fig. 8c). Choroid plexus carcinoma models driven by *Rb* KO, on the other hand, displayed a multitude of recurrent gains and losses that generally recapitulated the human disease (Extended Data Fig. 8d). H3.3(G34R)/PDGFRA-driven models were characterized by different chromosomal losses, the most recurrent being loss of chromosomes 18 and 19. This matched the human disease, which was also dominated by chromosomal losses, about half of which (chromosomes 5, 9, 10 and 18) contained regions syntenic to the two recurrent losses observed in mouse tumors (Fig. 5f). PHGG models featuring *Nf1* KO displayed varying CNAs but frequently harbored gain of chromosome 5, which was syntenic to the most frequent CNA observed in human NF1-driven pHGG, gain of chromosome 7 (*P* = 0.046; Fig. 5g).

Notably, we analyzed three different subgroups of mouse IHG-related models, driven by ALK, NTRK and ROS1, respectively, which have previously been stratified into the same tumor category^13,23^. We found that ALK-, NTRK- and MET-driven tumors displayed highly recurrent losses of chromosome 4, which overlapped with most of the observed losses in a relatively low number of available human cases (Fig. 5h,i and Extended Data Fig. 8e). Despite this clear overlap, statistical analysis indicated that the synteny between mouse chromosome 4 loss and the human summary plots was either not significant (ALK-driven pHGG) or barely significant (NTRK2-driven pHGG; *P* = 0.02), as a result of the small human patient population harboring chromosomal aberrations. Although this was also true for MET-driven pHGG, the number of available patient samples was even smaller (*n* = 6) and so did not allow for a meaningful comparison to mouse CNAs. By contrast, ROS1-driven models showed recurrent gain of chromosome 8, which was also syntenic to the most frequent gain in human ROS1-driven IHG (*P* = 0.048; Fig. 5j), indicating that these tumors likely represent a more distinct subset.

Whereas differential clustering of RTK-driven pHGG was also observed in our UMAP analyses (Extended Data Fig. 2k), human IHG have been shown to cluster more homogeneously^13^; we recapitulated this using the human datasets described here (Extended Data Fig. 8f).

Our results highlight that specific CNAs arise reproducibly in mouse brain tumors and that these are often syntenic to human counterparts.

### Reproducible tumor evolution during in vivo passaging

**Fig. 6:**
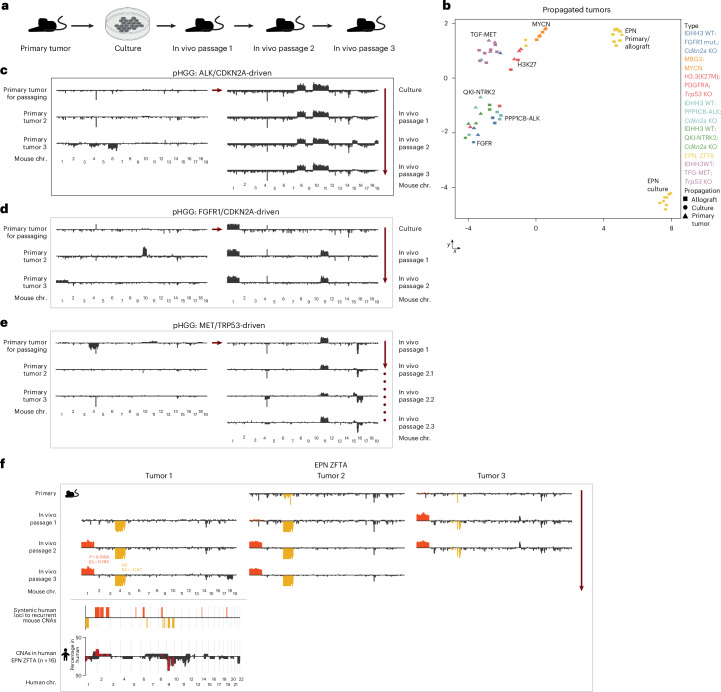
Continuous specific CNA evolution in mouse models. **a**, Scheme displaying propagation through allografting. **b**, UMAP of the 10,000 most significantly differentially methylated CpG sites of primary tumors, as well as allografts and/or cultured cells of the respective models. Most propagated samples showed a close similarity with their respective primary tumor. **c**–**e**, CNA profiles of ALK/CDKN2A-driven pHGG (**c**), FGFR1/CDKN2A-driven pHGG (**d**) and MET/TRP53-driven pHGG (**e**) revealed continuous acquisition of CNAs during passaging, including recurrent gain of chromosome 11. **f**, Three independently propagated allograft lines of the ZFTA-RELA ependymoma model ultimately all acquired a gain of chromosome 1 and loss of chromosome 4, indicating highly specific tumor evolution during in vivo passaging. Recurrent changes are color-coded. Lower section shows human CNA summary profiles of the indicated human tumor entity. The overlapping changes between mice and human are depicted in red. Syntenic overlap of each color-coded mouse CNA with human summary plots was statistically analyzed as indicated by *P* values in the same color. Enrichment scores were calculated by comparing the overlap of the indicated CNA with the corresponding human tumor entity to the overlap of that CNA with a pediatric reference set. Illustration in **a** created using BioRender.com.

We then investigated the stability of mouse methylation profiles over multiple in vivo passages (Fig. 6a). We initially clustered all allografts, respective primary tumors and intermediate in vitro cultures and found that all samples largely grouped with their respective entity (Fig. 6b). However, copy number profiles of these samples indicated continuous tumor evolution during in vivo passaging (Fig. 6c–e). We considered whether this evolution was directed and reproducible and analyzed the copy number profiles of three independently propagated tumor lines of the same RELA-driven ependymoma model (Fig. 6f). Not only did these lines ultimately acquire the same genomic alterations, the resulting CNAs were also in large agreement with those of respective primary human tumors (Fig. 6f). This suggests that for distinct entities, the copy number profiles of later in vivo passages might more closely reflect endpoint patient tumors than primary mouse cancers, potentially owing to a more similar timespan of tumor evolution.

**Extended Data Fig. 9:**
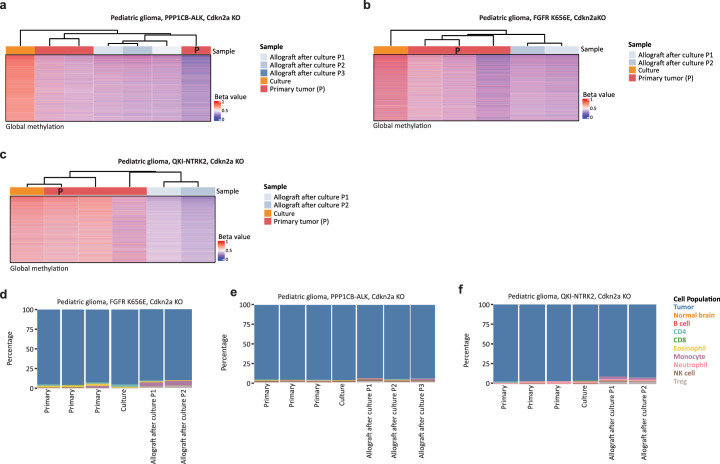
Characteristics of allografts and in vitro cultivated tumors. **a**–**c**, Hierarchical clustering of global DNA methylation profiles (10,000 most significantly differentially methylated CpGs) of indicated samples. In the PPP1CB-ALKand FGFR K656E-driven model, the primary tumors that were used for in vitro and in vivo propagation (P) cluster closer to allografts than to the intermediate in vitro culture. **d**–**f**, The immune cell infiltration is comparably low in cultivated as well as in allografted mouse tumors, allowing comparison of methylation profiles.

Of note, despite this continuous genetic evolution, allografts that derived from in vitro cultures clustered overall closer to primary tumors than to in vitro cultures, indicating that tumor cells reacquire certain methylation marks when grown in an in vivo environment (Extended Data Fig. 9a–c). To ensure that this effect was not an artifact of infiltrating immune cells that were only present in vivo, we analyzed the immune composition of all allografts but found only minimal levels of immune and normal brain cells (Extended Data Fig. 9d–f).

## Discussion

Here we analyzed the methylomes of 106 globally available mouse models for pediatric solid tumors, of which 18 pHGG models were generated within this study, including multiple IHG/ped. RTK models with RTK fusions. We found by UMAP analysis of methylation profiles that ROS1- and MET-driven tumors clustered differentially compared to NTRK- and ALK-driven models, and ROS1-driven pHGG displayed distinct CNAs. Although we recapitulated these differences in human CNA summary plots, our results, as well as previously published data, show that human IHG cluster more homogeneously^13,23^. This discrepancy could indicate different mechanisms of tumorigenesis that involve different CNAs in the two species, whereas the cell-of-origin pool could be more diverse in mice than in humans, leading to different UMAP clusters.

We established a workflow to integrate data from the Illumina Mouse Methylation BeadChip with data from the widely used human EPIC array. Our number of 675 identified probes was in line with a previous report that identified 255 cross-species tissue-specific CpG sites^67^. We used this workflow to validate the resemblance of mouse models to human counterparts and to confirm previously reported relationships, for example, between grade 3 MB and the pineal gland^56,57^. Of 81 analyzed mouse tumor samples, 25 did not match the respective human tumor family and 51 did not match the specific subtype using either of our analysis methods. Future studies may elucidate this incongruity further, as potential causes are likely to be sample specific and range from normal cell contamination, methodological limitations and modeling flaws to differences between murine and human tumor biology.

We also established a mouse immune cell methylation reference matrix, utilized it to deconvolute mouse tumor samples and validated our findings using IHC and comparison to human tumors. Our results recapitulated those of similar studies performed with human tumors that displayed varying amounts of monocytes and no neutrophil infiltration in glioma^64,68^. We found that SHH MB were not predicted to contain monocytes, T_reg_ cells or NK cells, consistent with a previous study in which low levels of monocytes but no T_reg_ cells or NK cells were identified in human samples^64^. Notably, we found a uniquely prevalent population of B cells in SHH MB, which was also observed in human SHH MB and in other human brain tumor entities^64^. These results show the immune compositions of various pediatric mouse models.

Previous reports have described how DNA methylation artificially changes during two-dimensional cell culture and raised the importance of using serum-free three-dimensional culturing conditions^69–71^. Here we show that tumor spheres grown in serum-free media also undergo epigenetic changes. However, allografts derived from these spheres regained certain in vivo characteristics, suggesting that allografting of tumor cells may provide reliable models even after short-term in vitro culture. Of note, the two models in which the primary tumor clustered closest to allografts (ALK- and FGFR1-driven) had been cultured for eight and nine passages, respectively, whereas NTRK-driven tumor cells were only in culture for six passages.

Finally, we utilized our methylation atlas to investigate CNAs. Although distinct CNAs have been extensively described in various cancer entities^7–9^, and their importance in tumorigenesis has been increasingly appreciated during recent years^4–6,66^, there have so far been very limited options to interrogate the role of CNAs during tumor initiation, owing to missing model systems^4–6^. Custom induction of CNAs using genome-editing tools is not yet efficient and specific enough and/or has not been tested sufficiently to induce CNAs reliably^72–74^. In contrast to CNAs, single nucleotide variants are well-characterized drivers of tumorigenesis that also arise during tumor evolution^75^ and can be readily induced using modern genetic engineering tools.

Here we make four seminal CNA-related observations. (1) CNAs are highly recurrent in mouse models for pediatric solid tumors. This suggests that CNAs, in addition to the induced oncogenic hits, are also an essential part of tumorigenesis in mice. Although their importance has been recognized in patient samples with more heterogenous CNA landscapes^4–6^, it was not clear that mouse tumors with strong drivers such as *MYC* overexpression and *Trp53* KO would also cooperate with distinct CNAs during tumorigenesis. (2) CNAs are tumor-type specific or even oncogene specific, consistent with highly specific and complex molecular tumor development. Nearly every CNA pattern that we observed in our mouse model cohort was unique to the respective tumor subentity, indicating that we are far from having understood the intricate mechanisms of individual tumorigenesis. There is ongoing debate regarding the optimal degree of granularity in molecular subgroupings of pediatric tumors^76–78^. If the high recurrence of CNAs reflects essential, subgroup-specific roles in tumor formation, it would be reasonable to assume that the combination of oncogenic hits and CNAs within a distinct developmental cell population may constitute the minimal basis for pediatric tumor stratification. Together with an increasingly appreciated role of CNAs in tumorigenesis^4–6,66^, our results may shape future, highly detailed and CNA-informed tumor classification as an essential step toward individualized therapies. (3) The CNAs that occur in mice have syntenic regions to CNAs that occur in their respective human counterparts, highlighting the fidelity of mice as a model organism for human cancer. Most importantly, this finding also qualifies the presented mouse models as the required tool to research CNAs when they arise in a tumor-specific context, which has not been possible using other models such as human endpoint tumors. (4) We further observed directed CNA evolution during multiple in vivo passages, complementing previous reports showing that CNAs are frequently early events but can also occur during later stages of tumor development^6,79^. We found that some recurrent CNAs matching the human disease occurred only after in vivo passaging, potentially owing to a prolonged tumor evolution that is more similar to that of patient tumors. This indicates that for some entities, allografts might recapitulate the human disease even more faithfully than primary mouse tumors.

Future studies derived from these results may include single-cell sequencing approaches to explore CNA acquisition in situ or CNA-related genetic manipulation of tumors cells. Furthermore, our CNA data may help to narrow down key genes of interest from broader human CNAs through analysis of the minimal syntenic overlap; this would greatly advance our understanding of the players involved in entity-specific tumorigenesis. In the future, the identified CNAs could help to answer broader biological questions pertaining to human–mouse synteny or gene function.

In summary, we have presented a methylome atlas for mouse models of pediatric solid cancers and utilized it to characterize new and available models regarding their similarity to human tumors and microenvironmental compositions. Most notably, we identified highly recurrent and distinct CNA evolution in mouse tumors, enabling future work to determine the role of CNAs during tumorigenesis.

## Methods

### Mouse models

Mouse tumor samples of published mouse models of pediatric solid tumors were collected from various laboratories^10,13,16–18,29–50^. New pHGG models were generated by in utero electroporation of CRISPR–Cas9 and Tol2-transposon constructs, or by orthotopic transplantation as previously described^13,16^. Briefly, plasmids encoding the desired hit combinations were electroporated into the developing brains of E14.5 mice (originally ordered from Janvier or Charles River) in utero. Successful electroporation was validated by postnatal luciferase imaging, and only mice with a luciferase signal in the brain were kept until onset of symptoms or 1 year of age. In addition, we included tumor samples of a new genetically engineered mouse model for choroid plexus carcinoma, which will be presented elsewhere in more detail (*hGFAP-cre::lsl-MYCN,TP53*^*Fl/Fl*^*,lsl-Gli2*). All animal protocols for model generation, including allografts, were approved by the relevant authority (Regierungspräsidium Karlsruhe) under registration numbers G-168/17 and G-265/21, or by the state of Hamburg (reference N2019/99) and adhered fully to institutional guidelines. Animals were kept at ~22 °C and ~50% humidity. Dark/light cycles alternated between 20:00–06:00 and 07:00–19:00, respectively, with 1-h transition phases. All animals were sacrificed as soon as neurological symptoms were visible. If no symptoms were detected, animals were sacrificed after 1 year of lifespan or transplantation, and their brains were macroscopically investigated for signs of tumor growth after a sagittal cut along the midline. Cell culture conditions for tumor propagation were as previously described^13^. Hematoxylin and eosin and IHC staining were performed according to standard protocols as previously described^13^. The following antibodies were used: GFAP (GA5, 1:200, MAB3402, Sigma/Merck), Olig2 (1:100, ABE1024, Merck), CD20 (E3N7O, 1:100, 70168, Cell Signaling), CD3 (SP7, 1:75, ab16669, Abcam), Iba-1 (1:500, 019-19741, WAKO), anti-HA Tag (C29F4, Lot: 11, 1:1,000, 3724S, Cell Signaling)

### Sorting of immune cell populations

For FACS of immune cell populations, spleen and lymph nodes from CD-1 or C57BL6 mice were dissected and homogenized through a 40-μm filter into phosphate-buffered saline with 2% fetal calf serum using the plunger of a syringe. Erythrocyte lysis was performed for 5 min using ACK buffer (Lonza) at room temperature. After washing and centrifugation for 5 min at 350*g*, cells were stained with the corresponding antibodies: CD19 BUV395 (1D3, 1:300, 563557, BD), LIVE/DEAD Blue (1:500, L34961, ThermoFisher), CD11b BUV805 (M1/70, 1:500, 568345, BD), Siglec H Pacific Blue (551, 1:100, 129609, BioLegend), Ly6C BV510 (HK1.4, 1:500, 128033, BioLegend), NK1.1 BV785 (PK136, 1:400, 108749, BioLegend), TCR-β FITC (H57-597, 1:500, 109205, BioLegend), Ly6G PerCP-Cy5.5 (1A8, 1:500, 127615, BioLegend), CD127 PE (eBioSB/199, 1:50, 12-1273-82, eBioscience), CD335 NKp46 PE/Dazzle 594 (29A1.4, 1:300, 137629, BioLegend), CD4 PE-Cy7 (GK1.5, 1:500, 15-0041, eBioscience), CD25 APC (PC61, 1:200, 102012, BioLegend), CD8 Alexa Fluor 700 (53-6.7, 1:500, 56-0081-82, eBioscience), CD45 APC efluor780 (30-F11, 1:200, 56-0451, eBioscience).

The staining buffer was PBS with 2% FCS and 0.5 mM EDTA (FACS buffer). Subsequently, cells were washed using FACS buffer, centrifuged for 5 min at 350*g* and resuspended in 500 µl FACS buffer. Finally, cells were filtered through a 35-40-µM strainer, and immune cell populations were sorted using a FACSAria II (Becton Dickinson) equipped with a 70-µm nozzle based on their expression of the following markers: B cells: CD45^+^CD19^+^TCRb^−^; CD4^+^ T cells: CD45^+^CD19^−^ TCRb^+^CD4^+^CD8-; CD4^+^ T_reg_ cells: CD45^+^CD19^−^TCRb^+^CD4^+^CD8^−^CD127^−^CD25^+^; CD8^+^ T cells: CD45^+^CD19^−^TCRb^+^CD4^−^CD8^+^; NK cells: CD45^+^CD19^−^TCRb^−^CD11b^dim^NKp46^+^NK1.1^+^; granulocytes: CD45^+^CD19^−^TCRb^−^CD11b^+^Ly6G^+^Ly6C^−^; monocytes: CD45^+^CD19^−^TCRb^−^CD11b^+^Ly6G^−^Ly6C^+^; eosinophils: CD45^+^CD19^−^TCRb^−^CD11b^+^SiglecF^+^ SSC^high^.

### DNA extraction and sequencing

To generate DNA methylation profiles, DNA was extracted from mouse tumor tissue. The tumor tissue was dissected based on macroscopical examination and freshly frozen at −80 °C or cryopreserved. In addition, formalin-fixed paraffin-embedded (FFPE) tissue was used. Tumor tissue was extracted from paraffin blocks based on histological staining of adjacent slices. Standard kits and reagents were used according to the respective manufacturers’ protocols. DNA was bisulfite converted using an EZ DNA Methylation Kit (Zymo Research). For FFPE samples, a restoration kit (Infinium HD FFPE DNA Restore Kit, Illumina) was employed. Finally, 100–500 ng of DNA was applied to the 285k mouse chip array (Illumina), which was run on an iScan device (Illumina).

### Data analysis

All data analyses were performed in R (v.4.1.1). Plots were generated using ComplexHeatmap, umap, circlize, ggplot2 and ggalluvial. The tableau10 color scheme was used. Figures and schemes were generated with Adobe Illustrator (v.28.5). The idat files resulting from iScan were used for all analyses. First, a quality filter was applied. The SeSaMe package (v.1.12.9) function sesameQC was used, and the number of missing values, the mean intensity and the probe success rate of each sample were identified. The 95% percentile of each of the parameters was determined. Samples in the 5% outliers of all three parameters were excluded. The remaining samples were used for further analyses.

The processing of idat files was performed with SeSaMe (v.1.12.9) and the openSeSaMe pipeline, which generates normalized beta values. All comparisons were performed by identifying the 10,000 most significantly differentially methylated CpG sites. These high-dimensional data were visualized with UMAP (v.0.2.10.0). The limma package (v.3.58.1) was used to calculate significant CpG sites per group (conditions: adjusted *P* < 0.05, |log fold change| > 0.2). The CpG sites located in the promoter region of a gene were determined using https://github.com/zhou-lab/KYCG_knowledgebase_MM285 and then displayed as a heatmap with ComplexHeatmap (v.2.18.0). Human rhabdoid tumor DNA methylation data for Fig. 2e were derived from GSE123601, GSE109381 and GSE228091.

### Transcriptome comparison

For correlation of the mouse methylome with the transcriptome, publicly available transcriptomic data of different AT/RT, MRT, ETMR and MB mouse models were analyzed in R Studio (v.4.4.1). Raw data were downloaded from GSE188654, GSE103348, GSE112699, GSE120344, GSE107263, GSE155471, GSE65888, GSE62625, GSE24628, GSE50824 and GSE2426. In addition, mouse MRT data were supplied by R. Imle, and data from *Gli2*-altered SHH MB were provided by Y. Pei.

For gene expression array data, each dataset was *z*-score normalized. VST-normalization, included in the DESeq2 package^80^, was performed for all bulk RNA sequencing datasets separately, with subsequent *z*-score normalization. Data were merged, and batch effects resulting from different source laboratories and array types were corrected using ComBat_seq(), also included in DESeq2. The top 1,000 most variable genes among all samples were identified, and the results were visualized using the umap package (v.0.2.10.0^81^). To find differentially expressed genes between each entity and/or subgroup, the difference in *z*-scores for each gene was calculated. The top differentially expressed and methylated genes in mouse AT/RT MYC versus AT/RT SHH were exemplarily correlated with human bulk RNA sequencing data^82,83^ (AT/RT SHH: *n* = 18; AT/RT MYC: *n* = 9). Differentially expressed genes for the human samples were identified as above, and results were visualized in a volcano plot using the function EnhancedVolcano() of the EnhancedVolcano package (10.18129/B9.bioc.EnhancedVolcano) with annotation of the genes identified in the mouse data.

### Immune cell deconvolution

To determine the proportion of infiltrating immune cells, we used a deconvolution approach as described for human tumors by Grabovska et al.^64^. First, a reference matrix was constructed using the DNA methylation profiles of pure immune cell populations, the mean of a representative set of mouse tumors and the mean of normal mouse brain samples. Of these, the significantly differential CpG sites of each population versus all others were determined using limma (v.3.50.3). Then, all sites with a significant *P* value (<0.05) were retained for further use, and the 100 sites with the highest delta-beta values were selected. Thereby, a reference matrix of 1,000 CpG sites was generated (Supplementary Table 7). This reference matrix was then used to deconvolute immune, tumor and normal cell populations with the Epidish package (v.2.10.0) using default settings.

In addition, immune cell deconvolution was performed using PRmeth (10.1186/s12859-022-04893-7). Here two unknown cell populations and the eight sorted immune cell profiles were included for deconvolution. The deconvolution was performed with iters = 1,000, rssDiffStop = 1 × 10^−10^.

To evaluate our deconvolution approach, we also tested the reference matrix published by Schönung et al.^63^ and applied it to our sorted immune cell populations using EpiDish with default settings.

### Human–mouse comparison

We developed a workflow to compare the DNA methylation profiles of mouse tumor models and human brain tumors. First, a human methylation reference set was constructed from GSE109381. All 91 classes were included, with a maximum of 20 samples per class, followed by preprocessing with minfi (v.1.40.0). Noob preprocessing was applied, and confounding CpGs were excluded. Data were then quantile normalized, and the 15,000 most differentially methylated CpG sites were identified. For the mouse samples, all samples were processed individually, and the ortholog CpGs to the human EPIC array (described at https://zwdzwd.github.io/InfiniumAnnotation) were selected. The overlap of 675 CpGs was then used for all further analyses. The discrimination of human tumors by this set of CpG sites was confirmed by validating the stability of tumor groups. The *x* and *y* coordinates of the first five iterations of UMAP dimensionality reduction from the human dataset were calculated with the umap package (v.0.2.10.0). After randomly downsampling to 90%, we performed a pairwise correlation of the *x* and *y* coordinates for 500 iterations using stats (v.4.3.0).

The human reference set and the mouse sample were quantile normalized, and the methylation patterns were visualized in a UMAP. To account for the instability of UMAP visualizations, we generated 10 UMAPs per mouse sample with a randomly chosen 90% of human tumor samples. We then calculated the mean distance in UMAP coordinates between the mouse sample and each human brain tumor type. The most frequent nearest neighbor per model was used for the following matching analysis. In addition, we established a random forest classifier based on the quantile normalized beta values of the selected 675 CpG sites of the human reference data, which was trained using the randomForest package (v.4.7-1.2). To ensure robust model performance, the number of trees was set to 1,000. Class imbalance was addressed by setting the sampsize parameter to draw an equal number of samples from each class, corresponding to the size of the smallest class. This classifier was used to predict the class of each mouse sample. The most frequent hit per model was used for the following matching analysis.

To statistically test the validity of our comparisons, we performed a one-sided binomial test and used Benjamini–Hochberg false discovery rate correction to correct *P* values for multiple testing.

To compare the success of matching different mouse models to the mutational load of human tumor entities, we used data derived from Sturm et al.^22^ and calculated mutational load as the number of mutations per 130 genes included in the panel.

### CNA plot generation

The SeSaMe function for generating copy number plots (cnSegmentation) was employed with slight modifications based on the Conumee package for human data. Briefly, the intensity values of each CpG were normalized to the average of normal tissue controls included in this project. To this end, three references were generated for female, male and, separately, FFPE tissue. Finally, the values of neighboring CpG sites were combined in bins leading to reliable segmentation results.

Chromosomal instability was calculated by determining the average percentage of bins (genomic positions) with a copy number change of ±0.1 of the total number of bins.

### CNA synteny comparison

Genomic human regions syntenic to specific mouse chromosomes were derived using Cinteny^84^ (http://cinteny.cchmc.org/) and visualized using IGV^85^ and Affinity Designer by generation of custom *.seg files. Human summary plots were generated in IGV using combined segmentation files for the indicated sample sets and visually prepared using Affinity Designer. The methylation data that we used to generate human CNA summary plots were derived from our reference cohort and long-term global sample collection^55^ (https://www.molecularneuropathology.org). Our human data showed good consistency with other previously published datasets, for example, for G34-mutated HGG^9^ or MB^86^. To perform a statistical evaluation of mouse CNA profiles, we generated a human reference CNA set using three copy number profiles per entity from our in-house brain tumor set and determined gains and losses as changes above or below a threshold of 0.1 or −0.1, respectively, and determined the percentage gained or lost in the human genome per patient. We performed the same analysis for the target entity specifically. We then calculated the overlap between human syntenic bins of the respective mouse chromosome and genomic alterations in both human datasets and subtracted the percentage altered overall from the percentage overlap (enrichment score per patient). Last, we compared the differences between all patients within target and reference datasets using a one-sided *t*-test. Subtraction of average enrichment scores in the reference dataset from average enrichment scores in the target datasets resulted in the final enrichment scores depicted in Fig. 5.

### Statistics and reproducibility

No statistical method was used to predetermine sample size. No data were excluded from the analysis provided the described quality criteria were fulfilled. The investigators were not blinded to allocation during experiments or outcome assessment.

### Reporting summary

Further information on research design is available in the Nature Portfolio Reporting Summary linked to this article.

## Online content

Any methods, additional references, Nature Portfolio reporting summaries, source data, extended data, supplementary information, acknowledgements, peer review information; details of author contributions and competing interests; and statements of data and code availability are available at 10.1038/s41588-025-02419-4.

## Supplementary information

Reporting Summary

Supplementary Tables Supplementary Tables 1–8.

## Data Availability

All data necessary for the conclusions of the study are provided with the article. All mouse DNA methylation data employed in this project can be found at GEO (GSE275151). Human rhabdoid tumor DNA methylation data were downloaded from GSE123601, GSE109381 and GSE228091. Transcriptomic data of different mouse models were downloaded from GSE188654, GSE103348, GSE112699, GSE120344, GSE107263, GSE155471, GSE65888, GSE62625, GSE24628, GSE50824 and GSE2426. Source data underlying all graphical representations used in the figures are provided as Supplementary Tables 1–8. Plasmids and engraftable tumor cells of new models for pHGG will be shared with the scientific community upon request to the corresponding author.

## Code availability

No custom code or software was generated within this work. All information regarding software packages and versions can be found in Methods.
