# Supplementary material for: Investigation of a global mouse methylome atlas reveals subtype-specific copy number alterations in pediatric cancer models
Source: Nat Genet. 2025 Dec 11;58(1):143–56. doi: 10.1038/s41588-025-02419-4 (PMC12807868; doi:10.1038/s41588-025-02419-4)
Supplement: Supplementary file 1 — Reporting Summary [file 41588_2025_2419_MOESM1_ESM.pdf]

nature portfolio

Corresponding author(s): Marc Zuckermann

Last updated by author(s): Aug 4, 2025

Reporting Summary

Nature Portfolio wishes to improve the reproducibility of the work that we publish. This form provides structure for consistency and transparency in reporting. For further information on Nature Portfolio policies, see our Editorial Policies and the Editorial Policy Checklist.

Statistics

For all statistical analyses, confirm that the following items are present in the figure legend, table legend, main text, or Methods section.

n/a	Confirmed	
☐	☒	The exact sample size (*n*) for each experimental group/condition, given as a discrete number and unit of measurement
☐	☒	A statement on whether measurements were taken from distinct samples or whether the same sample was measured repeatedly
☐	☒	The statistical test(s) used AND whether they are one- or two-sided<br>*Only common tests should be described solely by name; describe more complex techniques in the Methods section.*
☐	☒	A description of all covariates tested
☐	☒	A description of any assumptions or corrections, such as tests of normality and adjustment for multiple comparisons
☐	☒	A full description of the statistical parameters including central tendency (e.g. means) or other basic estimates (e.g. regression coefficient) AND variation (e.g. standard deviation) or associated estimates of uncertainty (e.g. confidence intervals)
☐	☒	For null hypothesis testing, the test statistic (e.g. *F*, *t*, *r*) with confidence intervals, effect sizes, degrees of freedom and *P* value noted<br>*Give P values as exact values whenever suitable.*
☒	☐	For Bayesian analysis, information on the choice of priors and Markov chain Monte Carlo settings
☒	☐	For hierarchical and complex designs, identification of the appropriate level for tests and full reporting of outcomes
☐	☒	Estimates of effect sizes (e.g. Cohen's *d*, Pearson's *r*), indicating how they were calculated

Our web collection on statistics for biologists contains articles on many of the points above.

Software and code

Policy information about availability of computer code

Data collection	The idat files resulting from the iScan were used for all analyses. Initially, a quality filter was applied using the SeSaMe package (1.12.9) function “sesameQC”.
Data analysis	All data analyses were performed in R (4.1.1). Plots were generated by ComplexHeatmap, umap, circlize, ggplot2 and ggalluvial. Significantly different CpG sites were determined by limma (v3.58.1). The CpG sites located in the promoter region of a gene were determined using https://github.com/zhou-lab/KYCG_knowledgebase_MM285 and then displayed as a heatmap with ComplexHeatmap (v2.18.0). The Epidish package (2.10.0) and PRmeth (https://doi.org/10.1186/s12859-022-04893-7) were used for deconvolution approaches. Pre-processing of human referenc samples was done with minfi (1.40.0). For gene expression array data, the DESeq2 package (v1.46.0) and the EnhancedVolcano package (v1.24.0) were used. For mouse-human matching, we trained a random forest classifier using the randomForest package (v4.7-1.2). Data was visualized using IGV, Adobe Illustrator (28.5), UMAP (umap, v0.2.10.0) and Affinity Designer.<br><br>No custom code or software has been generated within this article.

For manuscripts utilizing custom algorithms or software that are central to the research but not yet described in published literature, software must be made available to editors and reviewers. We strongly encourage code deposition in a community repository (e.g. GitHub). See the Nature Portfolio guidelines for submitting code & software for further information.

Data

Policy information about availability of data

All manuscripts must include a data availability statement. This statement should provide the following information, where applicable:

- Accession codes, unique identifiers, or web links for publicly available datasets
- A description of any restrictions on data availability
- For clinical datasets or third party data, please ensure that the statement adheres to our policy

All data necessary for the conclusions of the study are provided with the article. All mouse DNA methylation data employed in this project can be found under GEO (GSE275151). Human rhabdoid tumor DNA methylation data has been downloaded from GSE123601, GSE109381 and GSE228091. Transcriptomic data of different mouse models were downloaded from GSE188654, GSE103348, GSE112699, GSE120344, GSE107263, GSE155471, GSE65888, GSE62625, GSE24628, GSE50824 and GSE2426. Source data underlying all graphical representations used in the figures are provided as Supplementary Tables. Plasmids and engraftable tumor cells of novel models for pHGG will be shared with the scientific community upon request.

Research involving human participants, their data, or biological material

Policy information about studies with human participants or human data. See also policy information about sex, gender (identity/presentation), and sexual orientation and race, ethnicity and racism.

Reporting on sex and gender	The study does not involve human participation.
Reporting on race, ethnicity, or other socially relevant groupings	The study does not involve human participation.
Population characteristics	The study does not involve human participation.
Recruitment	The study does not involve human participation.
Ethics oversight	The study does not involve human participation.

Note that full information on the approval of the study protocol must also be provided in the manuscript.

Field-specific reporting

Please select the one below that is the best fit for your research. If you are not sure, read the appropriate sections before making your selection.

☒ Life sciences ☐ Behavioural & social sciences ☐ Ecological, evolutionary & environmental sciences

For a reference copy of the document with all sections, see nature.com/documents/nr-reporting-summary-flat.pdf

Life sciences study design

All studies must disclose on these points even when the disclosure is negative.

Sample size	Every mouse model available to us was included in the study, if possible with n=3 mice per model.
Data exclusions	To ensure high-quality data, a quality filter was applied. Therefore, the SeSaMe package (1.12.9) function "sesameQC" was used and the number of NAs, the mean intensity as well as the probe success rate of each sample were identified. The 95% percentile of each of the parameters was determined. All samples were excluded, which were in the 5% outliers of all three parameters. The remaining samples were used for further analyses.
Replication	If available, three mice per models were analyzed, which generally showed expected similarities for biological replicates.
Randomization	The study does not contain any experiments where group allocation would be applicable.
Blinding	The study does not contain any experiments where blinding would be applicable.

Reporting for specific materials, systems and methods

We require information from authors about some types of materials, experimental systems and methods used in many studies. Here, indicate whether each material, system or method listed is relevant to your study. If you are not sure if a list item applies to your research, read the appropriate section before selecting a response.

Materials & experimental systems

n/a	Involved in the study
☐	☒ Antibodies
☒	☐ Eukaryotic cell lines
☒	☐ Palaeontology and archaeology
☐	☒ Animals and other organisms
☒	☐ Clinical data
☒	☐ Dual use research of concern
☒	☐ Plants

Methods

n/a	Involved in the study
☒	☐ ChIP-seq
☒	☐ Flow cytometry
☒	☐ MRI-based neuroimaging

Antibodies

Antibodies used

GFAP GA5 1:200 MAB3402 Sigma/Merck
Olig2 1:100 ABE1024 Merck
CD20 E3N7O 1:100 70168 Cell Signaling
CD3 SP7 1:75 ab16669 abcam
Iba-1 1:500 019-19741 WAKO
anti-HA Tag (C29F4) Lot: 11 1:1000 3724S Cell Signaling
CD19 BUV395 1D3 1:300 563557 BD
LIVE/DEAD™ Blue 1:500 L34961 ThermoFisher
CD11b BUV805 M1/70 1:500 568345 BD
Siglec H Pacific Blue 551 1:100 129609 BioLegend
Ly6C BV510 HK1.4 1:500 128033 BioLegend
NK1.1 BV785 PK136 1:400 108749 BioLegend
TCR β FITC H57-597 1:500 109205 BioLegend
Ly6G PerCP-Cy5.5 1A8 1:500 127615 BioLegend
CD127 PE eBioSB/199 1:50 12-1273-82 eBioscience
CD335 (NKp46) PE/Dazzle™ 594 29A1.4 1:300 137629 BioLegend
CD4 PE-Cy7 GK1.5 1:500 15-0041 eBioscience
CD25 APC PC61 1:200 102012 BioLegend
CD8 Alexa Fluor 700 53-6.7 1:500 56-0081-82 ebioscience
CD45 APC efluor780 30-F11 1:200 56-0451 ebioscience

Validation

https://www.cellsignal.com/products/primary-antibodies/ha-tag-c29f4-rabbit-mab/3724?srsltid=AfmBOopbrFOvwDuHuqqezF7Dwli92-vCBoZ97Se_KF83VLauH3c99816

https://www.sigmaaldrich.com/DE/de/product/mm/mab3402?mmredirect=1&utm_source=google&utm_medium=cpc&utm_id=12478270955&utm_campaign=%7Bcampaignname%7D&utm_content=119588671918&utm_term=&gad_source=1&gad_campaignid=12478270955&gclid=Cj0KCQjwndHEBhDVARIsAGh0g3BmZC6QAO61rxPQEj2ODF7mDBln48vmrmLr0wdvDB8I37kSkqWltcYaAlMaEALw_wcB

https://www.sigmaaldrich.com/DE/de/product/mm/abe1024?utm_source=google&utm_medium=cpc&utm_id=12478270955&utm_campaign=%7Bcampaignname%7D&utm_content=119588671918&utm_term=&gad_source=1&gad_campaignid=12478270955&gclid=Cj0KCQjwndHEBhDVARIsAGh0g3BHVZmnmAF175AjbKzb3mkUD3-X_DDutR9uBrf_R0lQBaOjktB6nJcaAgjxEALw_wcB

https://www.cellsignal.com/products/primary-antibodies/cd20-e3n7o-xp-rabbit-mab/70168?srsltid=AfmBOoq8YeVRXqwr2NQ7CDbU5aLYf5qL19StmZ_9zrmUa5-4ZiTrBorT

https://www.abcam.com/en-us/products/primary-antibodies/cd3-epsilon-antibody-sp7-ab16669?srsltid=AfmBOoo3_ymCTWJWHwEs1vhqqmpsQcldZHppnRy_V2TkhHmPOYS5QIFL

https://labchem-wako.fujifilm.com/us/product/detail/W01W0101-1974.html

https://www.bdbiosciences.com/en-pl/products/reagents/flow-cytometry-reagents/research-reagents/single-color-antibodies-ruo/buv395-rat-anti-mouse-cd19.563557?tab=product_details

https://www.fishersci.de/shop/products/live-dead-fixable-blue-dead-cell-stain-kit-uv-excitation-3/15582241?srsltid=AfmBOoqB8-RJ8RrXcKNwtzjUmIQIMPBcP9sJjcgwrfnK7H5KnW208vY4

hhttps://www.bdbiosciences.com/en-dk/products/reagents/flow-cytometry-reagents/research-reagents/single-color-antibodies-ruo/buv805-rat-anti-cd11b.568345?tab=product_details

https://www.biolegend.com/de-de/products/pacific-blue-anti-mouse-siglec-h-antibody-6904

https://www.biolegend.com/de-de/products/brilliant-violet-510-anti-mouse-ly-6c-antibody-8726

https://www.biolegend.com/de-de/products/brilliant-violet-785-anti-mouse-nk-1-1-antibody-10367

https://www.biolegend.com/de-de/products/fitc-anti-mouse-tcr-beta-chain-antibody-270

https://www.thermofisher.com/antibody/product/CD45-Antibody-clone-30-F11-Monoclonal/56-0451-82

https://www.thermofisher.com/antibody/product/CD8a-Antibody-clone-53-6-7-Monoclonal/56-0081-82

https://www.biolegend.com/de-de/products/apc-anti-mouse-cd25-antibody-420?GroupID=BLG10428

https://www.thermofisher.com/antibody/product/CD4-Antibody-clone-GK1-5-Monoclonal/15-0041-82

https://www.biolegend.com/en-gb/products/pe-dazzle-594-anti-mouse-cd335-nkp46-antibody-13172?GroupID=BLG8849

https://www.antibodyregistry.org/AB_953562

https://www.biolegend.com/de-de/products/percp-cyanine5-5-anti-mouse-ly-6g-antibody-6116

Animals and other research organisms

Policy information about studies involving animals; ARRIVE guidelines recommended for reporting animal research, and Sex and Gender in Research

Laboratory animals	Mus musculus, NSG, CD1 and C57BL/6, up to 1.5 years, depending on the model and animal permit of the research institute
Wild animals	Study did not involve wild animals.
Reporting on sex	Sex has not been considered in this study.
Field-collected samples	Study did not involve samples collected in the field.
Ethics oversight	All animal protocols for model generation, including allografts, were approved by the relevant authority (Regierungspräsidium Karlsruhe) under registration numbers G-168/17 and G-265/21, or by the state of Hamburg (Reference N2019/99), respectively.

Note that full information on the approval of the study protocol must also be provided in the manuscript.

Plants

Seed stocks	The study does not comprise plants.
Novel plant genotypes	The study does not comprise plants.
Authentication	The study does not comprise plants.-
